# Mobile health-based physical activity intervention for individuals with spinal cord injury in the community: A pilot study

**DOI:** 10.1371/journal.pone.0223762

**Published:** 2019-10-15

**Authors:** Shivayogi V. Hiremath, Amir Mohammad Amiri, Binod Thapa-Chhetry, Gretchen Snethen, Mary Schmidt-Read, Marlyn Ramos-Lamboy, Donna L. Coffman, Stephen S. Intille

**Affiliations:** 1 Department of Health and Rehabilitation Sciences, Temple University, Philadelphia, Pennsylvania, United States of America; 2 Department of Electrical and Computer Engineering, Temple University, Philadelphia, Pennsylvania, United States of America; 3 Department of Biomedical Engineering, Widener University, Chester, Pennsylvania, United States of America; 4 Khoury College of Computer Sciences, Northeastern University, Boston, Massachusetts, United States of America; 5 Department of Health Sciences, Bouvé College of Health Sciences, Northeastern University, Boston, Massachusetts, United States of America; 6 Magee Rehabilitation Hospital, Jefferson Health, Philadelphia, Pennsylvania, United States of America; 7 MossRehab, Einstein Healthcare Network, Philadelphia, Pennsylvania, United States of America; 8 Department of Epidemiology and Biostatistics, Temple University, Philadelphia, Pennsylvania, United States of America; University of Alabama at Birmingham, UNITED STATES

## Abstract

Low levels of physical activity (PA) and high levels of sedentary behavior in individuals with spinal cord injury (SCI) have been associated with secondary conditions such as pain, fatigue, weight gain, and deconditioning. One strategy for promoting regular PA is to provide people with an accurate estimate of everyday PA level. The objective of this research was to use a mobile health-based PA measurement system to track PA levels of individuals with SCI in the community and provide them with a behavior-sensitive, just-in-time-adaptive intervention (JITAI) to improve their PA levels. The first, second, and third phases of the study, each with a duration of one month, involved collecting baseline PA levels, providing near-real-time feedback on PA level (PA Feedback), and providing PA Feedback with JITAI, respectively. PA levels in terms of energy expenditure in kilocalories, and minutes of light- and moderate- or vigorous-intensity PA were assessed by an activity monitor during the study. Twenty participants with SCI took part in this research study with a mean (SD) age of 39.4 (12.8) years and 12.4 (12.5) years since injury. Sixteen participants completed the study. Sixteen were male, 16 had paraplegia, and 12 had complete injury. Within-participant comparisons indicated that only two participants had higher energy expenditure (>10%) or lower energy expenditure (<-10%) during PA Feedback with JITAI compared to the baseline. However, eleven participants (69.0%) had higher light- and/or moderate-intensity PA during PA Feedback with JITAI compared to the baseline. To our knowledge, this is the first study to test a PA JITAI for individuals with SCI that responds automatically to monitored PA levels. The results of this pilot study suggest that a sensor-enabled mobile JITAI has potential to improve PA levels of individuals with SCI. Future research should investigate the efficacy of JITAI through a clinical trial.

## Introduction

Low levels of physical activity (PA) and high levels of sedentary behavior in 300,000+ individuals with spinal cord injury (SCI) in the US have been associated with secondary conditions such as pain, fatigue, weight gain, and deconditioning [1–4]. Tawashy *et al*. performed a cross-sectional survey-based study in individuals with SCI and found that higher levels of PA were correlated with lower levels of pain, fatigue, and depression [1]. In another article, Rimmer *et al*. presented a framework called Disability-Associated Low Energy Expenditure (EE) Deconditioning Syndrome, which suggests that individuals with a neuromuscular disability have higher rates of sedentary behavior predisposing them to severe deconditioning [2]. Further, the article proposes the implementation of light-to-moderate intensity PA to increase movement-based EE, which in turn can reduce deconditioning in this population. One strategy for promoting regular PA is to provide people with an accurate estimate of everyday PA level [5]. Thus, a number of studies have developed and validated sensor-based activity monitors for individuals with SCI to quantify movement of the individual, wheelchair movement, and physiological changes, both in the laboratory and under free-living conditions [6–10].

Garcia-Masso *et al*. found that the activity counts from a wrist-worn activity monitor (Actigraph GT3X) were highly correlated with the criterion EE measure during housework activities, arm-ergometry, and propulsion (correlation: r = 0.86) [8]. Kiuchi *et al*. found that the EE estimated by an upper-arm movement sensor (left upper arm R^2^ = 0.75, right upper arm R^2^ = 0.87) was similar to the EE estimated by a wrist sensor (left wrist: R^2^ = 0.86, right wrist: R^2^ = 0.68) during wheelchair propulsion on a treadmill [9]. Hiremath *et al*. developed a PA monitoring system that combined a wheel rotation sensor and a wrist-worn movement sensor to estimate EE for individuals with SCI who use manual wheelchairs for mobility [10]. Results indicated that the PA monitor system estimated EE with an error of less than 10% for various wheelchair-based PAs in laboratory and community settings. While availability of a validated activity monitor system for individuals with SCI in the community is important for supporting PA research with this population, there is a need to develop tools that can both track PA levels and provide PA interventions in the community. Furthermore, availability of consumer friendly devices that are accessible and can accurately capture community-based PA in individuals with SCI have a potential to improve their quality of life.

Building on our prior research [10], we have developed a just-in-time adaptive intervention (JITAI) that measures PA using mobile sensing and responds automatically. A JITAI is an intervention design that uses mobile health (mHealth) technology to deliver intervention components at appropriate times and contexts to support individuals’ health behaviors [11–14]. The framework used for this study is based on the Conceptual Model of JITAI Components proposed by Nahum-Shani *et al*. [12], which highlights four components that play an important role in designing JITAI-based interventions. The four components include: decision points, intervention options, tailoring variables, and decision rules. JITAIs often provide an intervention through a smartphone and/or a wearable activity monitor. The system determines that–based on automatically-measured behavior or context–a particular intervention component, such as prompting an individual to perform a PA, could be helpful or especially effective. Whether to deliver an intervention is determined by JITAI decision rules, which are if-then rules that are typically constructed based on individual’s prior PA behavior in the community.

Ginis *et al*. examined social cognitive theory variables as predictors of PA among individuals with SCI [15]. The research showed that self-regulation was a significant and direct predictor of PA; and self-regulatory efficacy and outcome expectations, mediated by self-regulation, had indirect effects on PA. The passive real-time feedback has the potential to make the individual self-aware of their PA levels. This approach may be effective in a small percentage of individuals who are very motivated to increase their PA levels. However, to achieve long-term engagement, which will be required to support long-term behavior change and maintenance, automatic detection of behavior may permit delivery of highly-tailored positive reinforcement and adaptive goal setting. The JITAI notifications will be based on the individual’s current PA levels and the context of the detected PAs over prior days. JITAI can incorporate operant conditioning [16], a learning technique that can increase or decrease the probability of a behavior (e.g. performing PA) by providing stimulus following the behavior [12]. For example, congratulatory or positive feedback messages to individuals when they attain their goal or increase their PA levels may reinforce those behaviors, making them more likely in the future. The objective of this study was to assess whether sensor-based, automatic tracking of light- and moderate- or vigorous-intensity PA of individuals with SCI in the community could be used to develop a JITAI used to increase PA levels. We hypothesized that such a JITAI would lead to an increase in light- and moderate-intensity PA in individuals with SCI.

## Methods

### Participants

**Fig 1:**
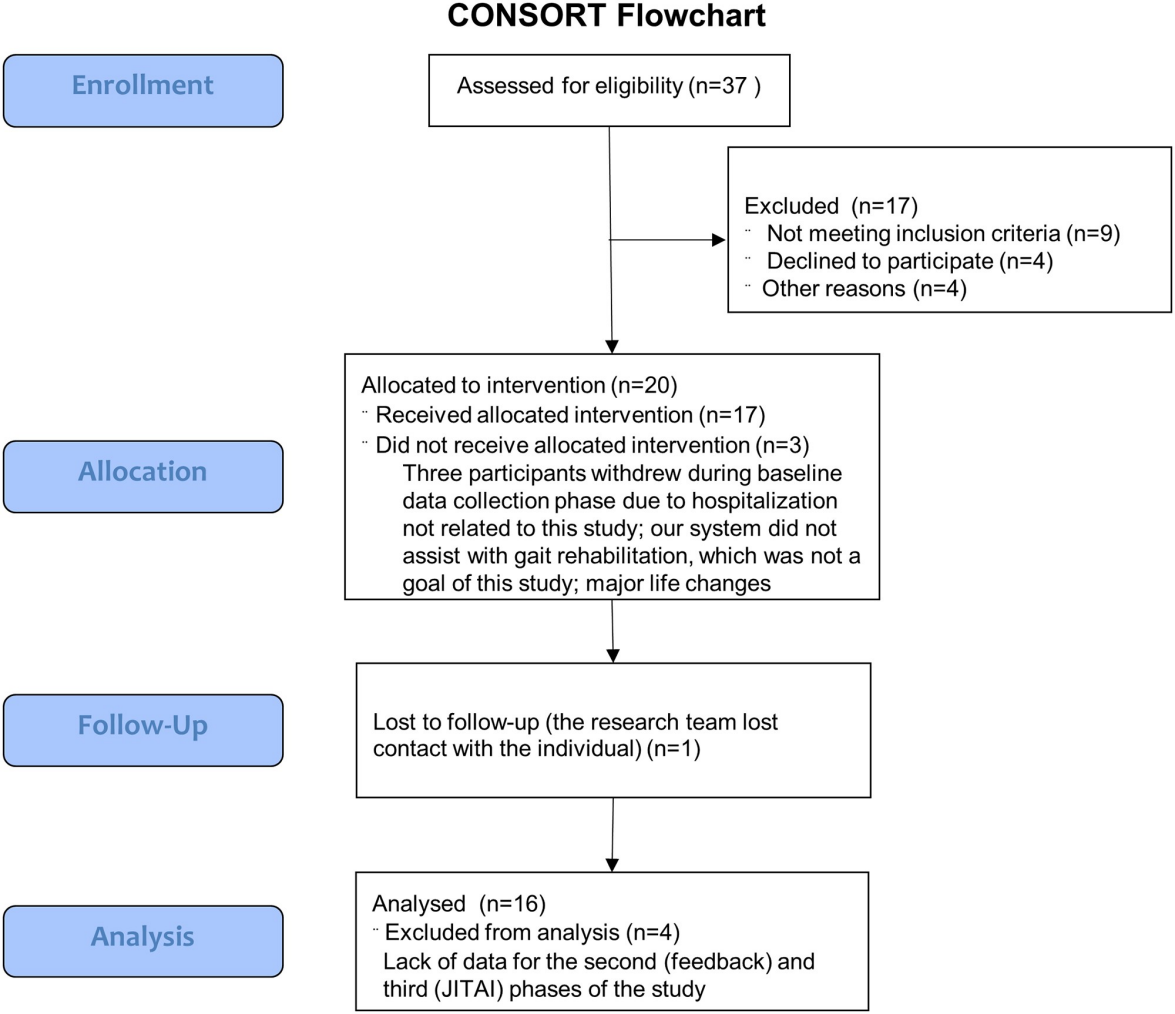
Consort Flowchart. Flow diagram of the progress of participants through the non-randomized pilot study.

A sample size of 20 participants was chosen based on budget constraints and other pilot studies [9, 17, 18]. Participants were included if they were 18–65 years of age, had a diagnosis of SCI (traumatic or non-traumatic), were at least 6 months post-injury, used a manual wheelchair as their primary means of mobility, self-propelled their wheelchair, were medically stable, and had experience using a smartphone. Participants were excluded if they had active pelvic or thigh wounds (pressure injuries), a history of cardiovascular disease, or were pregnant. The research team worked with a physiatrist from MossRehab, Einstein Healthcare Network, Philadelphia, and a physical therapist from Magee Rehabilitation Hospital, Philadelphia area to recruit participants for the study. Twenty of the 37 participants who were screened met the inclusion criteria and took part in the study (Fig 1). Participants lived within 90 miles of Philadelphia, USA. All participants provided a written informed consent, approved by Temple University’s Institutional Review Board (IRB), prior to their participation. The study was approved by Temple University, Northeastern University, Magee Rehabilitation Hospital, and Einstein Healthcare Network (S1 File). We certify that all applicable institutional regulations concerning the ethical use of human volunteers were followed during the course of this research. The study was retrospectively registered at ClinicalTrials.gov (NCT03773692) as we did not realize that this non-randomized pilot study of tracking PA levels and providing feedback and JITAI to participants fit the World Health Organization’s definition of a clinical trial.

### Instrumentation

**Fig 2:**
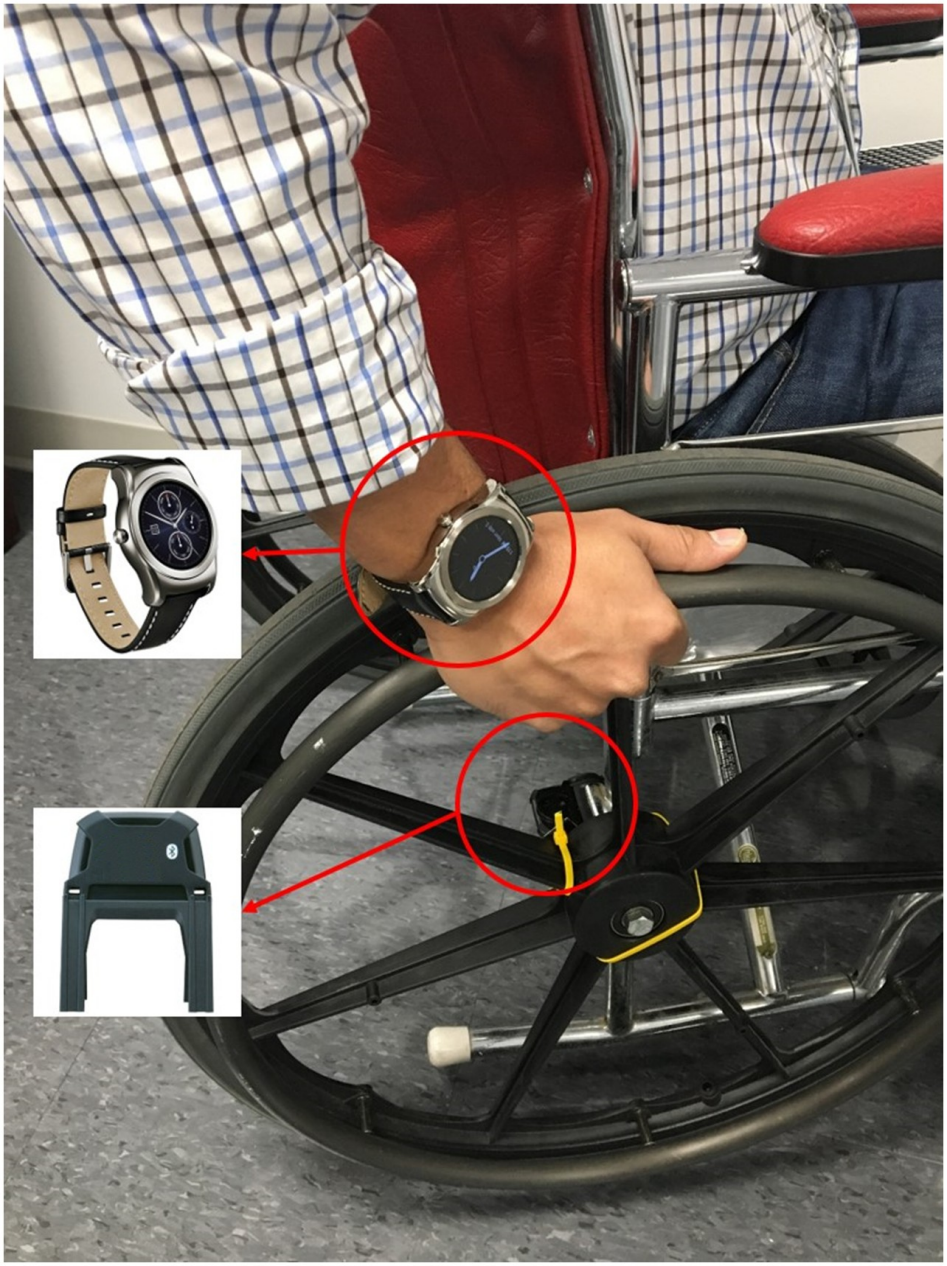
A participant using the JITAI sensors to measure and motivate PA.

The mobile JITAI used off-the-shelf components such as an Android-based smartphone (Nexus 5 or 5X, LG Corp., Englewood Cliffs, NJ, USA), a wrist-worn smartwatch (LG-Urbane, LG Corp., Englewood Cliffs, NJ, USA), and a Bluetooth-based wheel rotation monitor (PanoBike, Topeak Inc., Taichung, Taiwan) to detect wheelchair-based PAs in individuals with SCI (Fig 2). The smartwatch and wheel rotation monitor streamed data to the smartphone. Via the smartphone, the system also collected ecological momentary assessments (EMAs) about the type of PAs an individual was performing during the day for the duration of the study. The participants received six audio/vibration prompts, once every two hours, to answer questions on the smartphone (i.e., the EMAs), scheduled randomly from 9 am to 8 pm, or during a shifted 12-hour window more convenient for the participant. The choice of the EMA frequency was based on prior research in the general population [19, 20], which achieved a decent completion rate of 67%. The participants chose one of nine options for an activity they were performing at the moment the smartphone prompted. The nine options included ‘cellphone/tablet/TV,’ ‘resting,’ ‘pushing wheelchair,’ ‘eating,’ ‘in-transit,’ ‘arm-ergometry/hand cycling,’ ‘exercising,’ ‘sleeping,’ and ‘other activity.’ An unanswered EMA was prompted again after a delay of three and six minutes, respectively, if a participant was unable to respond to it the first or second time. These EMA responses allowed us to validate whether the PA a participant self-reported to be performing is detected by the smartphone application.

Participants wore the smartwatch, which records accelerometer data at 50 samples per second (50 Hz), on their preferred wrist. The raw accelerometer data were sent to the smartphone using the Bluetooth Low Energy (BLE) wireless protocol on an hourly basis. The wheel rotation monitor, consisting of a sensor and a magnet, were secured to the axle and spokes of a wheelchair wheel, respectively. Each wheel rotation was captured by the sensor and transmitted to the smartphone application in real-time using the BLE protocol. Statistical characteristics, known as features, were computed every minute from the raw data in real-time and sent to a smartphone application.

**Fig 3:**
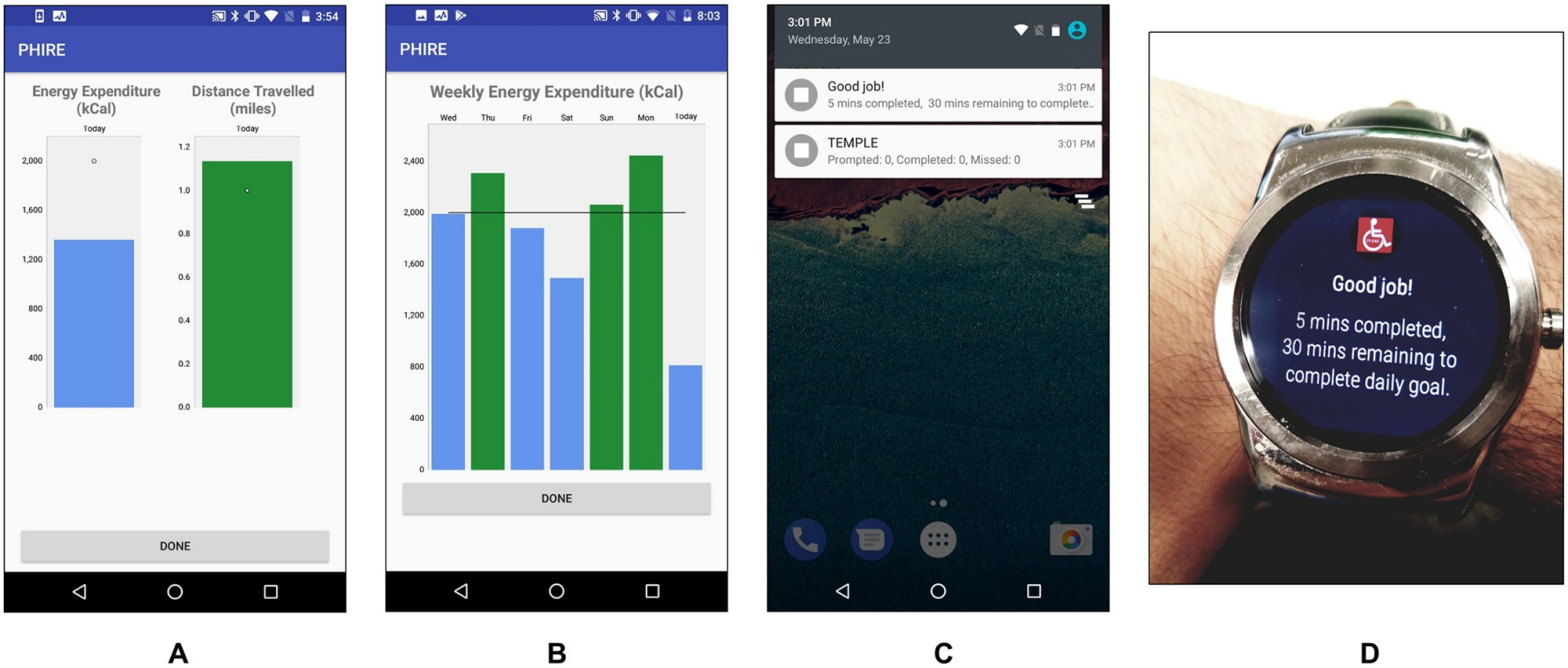
PA Feedback and JITAI provided by PHIRE app. (A) Daily and (B) weekly PA feedback were provided to the participants via the app during the 2^nd^ and 3^rd^ phases of the study. Examples of JITAI messages provided on the (C) smartphone and (D) smartwatch for the 3^rd^ phase of the study.

A smartphone application (app) called the Personal Health Informatics and Rehabilitation Engineering (PHIRE) app was developed and implemented for an Android-based smartphone (Fig 3). The PHIRE app used a C4.5 decision tree [21] machine-learning algorithm to classify wheelchair-based PAs and estimate PA levels once per minute [10]. The classification algorithm uses the real-time feature data from the smartwatch and wheel rotation monitor to detect wheelchair-based PAs. Wheelchair-based PAs include ‘resting,’ ‘arm-ergometry,’ ‘household activities,’ ‘activities that may involve some wheelchair movement,’ ‘wheelchair propulsion,’ ‘caretaker pushing,’ and ‘wheelchair basketball.’ In situations where the individual may not be performing a specific activity mentioned above (e.g. arm-ergometry), the classification algorithm will categorize the current PA into one of the seven wheelchair-based PA that best reflects the biomechanical movement pattern of the arm and/or wheelchair movement. The EE was estimated in a three-step process: 1) wheelchair-based PAs were detected in near-real-time, 2) the metabolic equivalent of a task (MET) for the wheelchair-based PA in individuals with SCI (paraplegia and tetraplegia) was obtained from a compendium listing activity-metabolic estimates [22], and 3) the EE was then estimated based on the METs and the weight of the participant [22]. The distance traveled in miles was calculated based on the wheelchair-wheel diameter and sensor reading from the wheel rotation monitor. All sensor data and logs were encrypted, saved on the smartphone, and uploaded to Google’s Firebase Cloud Storage on an hourly basis. Data were then downloaded to a desktop computer, decrypted, and viewed using custom desktop data visualization software. Research staff assessed each participant’s data, checking for existence of raw data and compliance on EMAs. Also available to the research team were data on smartphone and smartwatch memory, battery, and smartphone usage, which were helpful in interpreting missing data and estimating compliance of JITAI usage. The compliance of using JITAI was based on the duration of time the individuals wore the smartwatch, used the smartphone, and maintained the battery levels of both the devices. A research assistant contacted the participants if there were missing data for three consecutive days. While the PHIRE app needs to be rigorously tested in a large clinical trial prior to making it available to the public, we have made the code public via GitHub (link: https://github.com/binodthapachhetry/JustInTimeAdaptiveIntervention).

### Protocol

The study was conducted in three consecutive phases, each with a one-month duration. The first phase focused on collecting baseline PA level data. The second phase focused on providing near-real-time feedback on PA level (here onwards: PA Feedback) (Fig 3A and 3B). The third phase focused on providing PA Feedback with JITAI (here onwards: PA Feedback with JITAI) to the participants (Fig 3C and 3D).

#### First phase—Baseline data collection

Participants began the study by answering surveys related to demographics, SCI, wheelchair information, and health and PA history (S2 File). Participants also completed the Leisure Time Physical Activity Questionnaire for people with SCI (LTPAQ-SCI) [23], Fatigue Severity Scale (FSS) [24], and Wheelchair User’s Shoulder Pain Index (WUSPI) [25]. Prior research has validated the FSS [26], LTPAQ-SCI [23], and WUSPI [27–29] in individuals with SCI. These surveys provided us with information about the participant’s PA level and secondary conditions such as pain and fatigue.

Participants were provided with a smartphone, a smartwatch, and a wheel rotation monitor (Fig 2). Participants were asked to wear the smartwatch every day for the entire day, while keeping the smartphone in close proximity of the smartwatch. They were asked to charge the smartphone and smartwatch every night. Once sensor setup was completed, an investigator helped the participant install the PHIRE app on the smartphone and smartwatch by downloading it from the Google Play Store (Google LLC, Mountain View, CA, USA). Weight, height, age, gender, and injury level (paraplegia vs. tetraplegia) were entered into the PHIRE app. Participants were instructed to continue with their normal daily routine for one month.

#### Second phase—PA feedback

At the start of the second month, an investigator visited each participant’s home to collect survey information using the same instruments used at baseline. Post-survey completion, a PA recommendation handout based on PA guidelines for individuals with SCI was provided to the participants [3]. Participants were told to do at least 20 min of moderate-to-vigorous intensity aerobic activity, twice per week. They were also encouraged to do strength training exercises, consisting of three sets of eight to ten repetitions of exercise for each major muscle group, twice per week. The clinical recommendation was intended to help the participants improve their PA levels, health, and quality of life. Participants continued to use the same sensing equipment as in month one. The PHIRE app was updated to provide feedback about EE and distance traveled every minute during the day, and overall EE and distance traveled for the day and the week. Participants could view their feedback whenever they wanted, but were not prompted with the information (Fig 3A and 3B).

#### Third phase—PA feedback with JITAI

**Fig 4:**
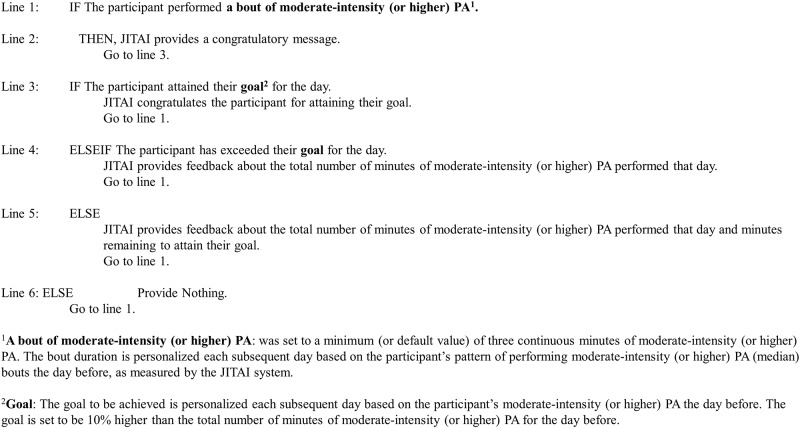
IF-Then rules used to design the JITAI notifications for the PA Feedback with JITAI phase of the study.

The third phase of the study was similar to the second phase of the study (i.e., the PA Feedback was available), but with an additional JITAI component. Post-survey completion, the PA recommendations were discussed again with the participants. The JITAI component of the study included providing proactively-prompted, near-real-time feedback through the smartphone (audio and/or vibration: based on participants’ choice) and smartwatch (vibration) when the participant performed a bout of moderate-intensity (or higher) PA. Fig 4 shows the IF-Then rules used to design the JITAI notifications. The default setting of the app was that a minimum of three continuous minutes of PA were observed before providing personalized feedback. Personalization was based on the participant’s prior patterns of conducting bouts of moderate-intensity PA, as measured by the system. Following this, the congratulatory messages were provided every minute until the participant stopped performing the moderate-intensity PA. Participants also received a congratulatory message when they reached and exceeded their daily goal. The daily goal was personalized each subsequent day based on the participant’s pattern of performing moderate-intensity PA the day before. The congratulatory message contained minutes of moderate-intensity PA performed and minutes remaining to attain their goal (Fig 3C and 3D).

At the end of the study an exit interview was conducted that asked the participants about the JITAI system’s ease of use, any challenges they faced with the system, what they thought about the PA level feedback and JITAI notifications, and if they would be interested in continuing to use the JITAI system and/or recommend it to their friends. Participants either provided a written response to the questionnaire or asked the help of an investigator to write their responses if they had functional limitations. In addition, they provided verbal responses to some questions that they felt needed more explanation. The verbal responses were transcribed by an investigator in real time and cross-checked with the other investigator who was present during the interview.

#### Data analysis

**Fig 5:**
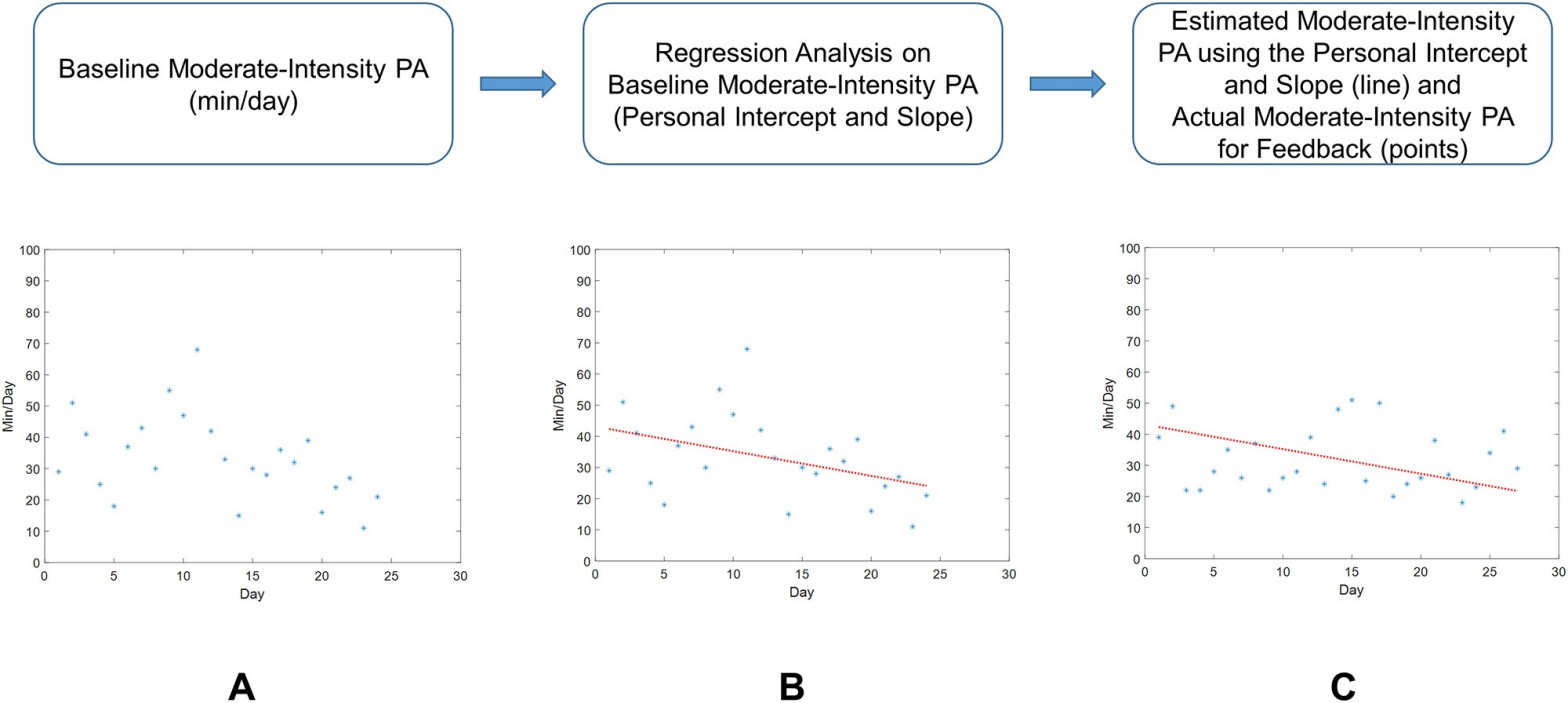
A data pipeline for within-participant analysis. (A) Baseline values of moderate-intensity PA per day over the course of first phase for one participant (ID#1), (B) Baseline data (points) were used to develop a regression model to obtain a fitted/predicted personal intercept and slope (continuous line) for the same participant. The regression line indicates a drop in moderate-intensity PA level over time, (C) Estimated moderate-intensity PA using personal intercept and slope (continuous line) from baseline (Fig 5B) and the moderate-intensity PA for PA Feedback phase (points).

Descriptive statistics including means and standard deviations were obtained for demographics, PA levels, pain, and fatigue. Baseline data (Fig 5A) was used to develop regression models for each participant and variable (EE, light-intensity PA, and moderate-intensity PA) to obtain a fitted/predicted personal intercept and slope (continuous line in Fig 5B). The personalized baseline regression model allowed us to account for a decrease or increase in the PA levels over the course of the study phase. Fig 5C shows the moderate-intensity PA for the estimated PA using personal intercept and slope (continuous line) from baseline (Fig 5B) and the moderate-intensity PA during PA Feedback phase (points). The value of PA data (EE in kcal, and light-intensity and moderate-intensity PA in min) for each day of PA Feedback (second phase), and PA Feedback with JITAI (third phase) were compared with the baseline (first phase) estimated using the person’s intercept and slope over time (Eq 1). Positive and negative percentage values represent an increase or decrease in PA levels compared to the baseline PA levels, respectively. The statistical significance for the change in PA levels was not assessed due to the small sample size and non-randomization of the participants to the second or third phases of this pilot study. The exit interview data–comprising of questionnaires answered and transcribed data–were analyzed by two investigators. The investigators reviewed the data to identify common themes from the data. Any differences in review results were discussed among investigators until consensus was reached.

$$\%Change\;in\;PA\;level=\frac{\text{PA}\;\text{level}\;\text{during}\;\text{PA}\;\text{Feedback}-\text{PA}\;\text{level}\;\text{for}\;\text{baseline}}{\text{PA}\;\text{level}\;\text{for}\;\text{baseline}}*100$$ (1)

## Results

### Participants

Twenty participants with traumatic SCI took part in this research study between July 2017 and November 2018. Participants were 16 males and 4 females with a mean (SD) age of 39.4 (12.8) years, 12.4 (12.5) years since injury, weight of 79.2 (14.3) kg and height of 1.8 (0.1) m. The SCI varied from cervical level three to thoracic level 12; twelve of the participants had a complete injury and 16 had paraplegia. Sixteen participants completed the study. Four participants withdrew from the study: the first participant withdrew after three weeks due to hospitalization not related to this study, the second participant withdrew after two days as our system did not assist with gait rehabilitation (which was not a goal of this study), the third participant withdrew after four weeks due to major life changes, and the fourth participant withdrew after six weeks as the research team lost contact with the individual. No adverse events were reported. Table 1 shows the mean (SD) leisure time PA collected every week over the course of the study for all participants who completed the study. Self-reported leisure time PA indicated that eight of the sixteen participants (50%) met the fitness guidelines for individuals with SCI during baseline phase [3] (20 min/day of moderate to vigorous-intensity PA twice a week). Table 2 shows the mean (SD) values for pain and fatigue collected every week over the course of the study for all participants how completed the study. Pain ranged from 0 to 57.1 (possible range: 0 to 150), indicating low to moderate interference of shoulder pain in performing regular activities. Fatigue severity ranged from 9 to 54.2 (possible range: 9 to 63), indicating no fatigue to high fatigue in the participants. The choice of the frequency of data collection using these surveys was based on the LTPAQ-SCI that measures self-reported PA for the last 7 days and the exploratory nature of this study.

**Table 1:** Mean (SD) values of leisure time PA across each phase of the study in the community. Self-reported leisure time PA collected using the LTPAQ-SCI.

ID#	Light-intensity PA in min/day			Moderate-intensity PA in min/day			Vigorous-intensity PA in min/day		
	Baseline	PA Feedback	PA Feedback with JITAI	Baseline	PA Feedback	PA Feedback with JITAI	Baseline	PA Feedback	PA Feedback with JITAI
1	27.4 (37.6)	0.9 (1.9)	1.1 (1.6)	15.4 (34.5)	7.7 (17.3)	1.1 (2.6)	0.0 (0.0)	0.0 (0.0)	0.0 (0.0)
3	25.7 (22.6)	34.6 (9.5)	33.4 (25.1)	27.4 (21.3)	32.1 (25.6)	17.1 (12.4)	12.0 (7.2)	30.5 (3.2)	27.9 (12.4)
4	36.4 (14.6)	49.7 (9.9)	58.9 (26.96)	10.7 (8.2)	24.9 (16.2)	80.4 (27.0)	0.0 (0.0)	0.0 (0.0)	0.0 (0.0)
5	37.6 (28.4)	90.0 (18.2)	78.6 (62.6)	33.6 (8.7)	60.0 (24.2)	37.8 (39.5)	39.9 (11.1)	27.9 (7.1)	28.2 (8.7)
6	9.4 (8.2)	10.0 (2.02)	5.7 (8.1)	15.7 (6.9)	12.9 (6.1)	0.0 (0.0)	10.7 (9.8)	0.0 (0.0)	0.0 (0.0)
7	12.7 (18.4)	10.5 (3.3)	13.6 (2.7)	21.6 (23.1)	9.1 (3.6)	6.6 (4.4)	4.1 (5.5)	4.3 (3.8)	3.2 (3.2)
8	0.0 (0.0)	8.1 (5.02)	0.0 (0.0)	0.0 (0.0)	2.9 (1.2)	0.0 (0.0)	0.0 (0.0)	1.4 (1.2)	0.0 (0.0)
9	62.1 (33.3)	47.1 (6.1)	24.3 (10.9)	34.3 (24.2)	51.4 (12.1)	31.1 (24.6)	34.3 (24.2)	49.3 (15.2)	23.4 (14.2)
10	20.7 (4.5)	12.2 (4.9)	13.7 (4.5)	5.7 (2.8)	8.6 (2.1)	8.8 (1.6	0.4 (0.7)	1.6 (0.7)	3.7 (2.1)
12	25.7 (18.2)	33.6 (8.66)	45.3 (4.2)	15.7 (2.9)	20.7 (4.5)	17.5 (2.3)	0.5 (1.1)	28.6 (21.6)	11.6 (11.8)
13	4.7 (6.8)	3.6 (3.6)	2.9 (4.3)	1.3 (2.9)	0.0 (0.0)	0.6 (1.1)	0.0 (0.0)	0.0 (0.0)	0.0 (0.0)
15	22.9 (26.2)	42.9 (8.6)	41.4 (11.4)	51.4 (37.4)	44.6 (15.3)	38.6 (20.1)	42.9 (39.3)	41.1 (15.3)	28.6 (14.0)
16	53.6 (8.2)	36.4 (16.2)	34.3 (24.2)	19.3 (22.5)	38.6 (31.7)	0.0 (0.0)	10.7 (12.9)	0.0 (0.0)	0.0 (0.0)
18	31.4 (17.0)	34.0 (15.2)	16.7 (15.3)	13.0 (2.7)	12.6 (1.6)	15.5 (5.5)	2.9 (2.0)	4.6 (1.2)	4.3 (1.4)
19	42.5 (52.0)	36.4 (55.8)	39.0 (47.1)	15.7 (18.7)	47.1 (52.1)	24.4 (12.4)	27.9 (30.8)	22.5 (8.3)	37.4 (29.4)
20	3.1 (1.9)	1.1 (1.3)	4.5 (4.8)	1.9 (1.9)	0.3 (0.6)	1.3 (1.3)	0.9 (1.9)	0.0 (0.0)	1.3 (2.9)
Group	26.0 (17.8)	28.2 (23.8)	25.8 (22.9)	17.7 (13.8)	23.3 (19.8)	17.5 (21.6)	11.7 (15.5)	13.2 (17.1)	10.6 (13.5)

**Table 2:** Mean (SD) values of pain and fatigue across each phase of the study in the community. Self-reported pain and fatigue were collected using the WUSPI and FSS instruments, respectively.

ID#	WUSPI				FSS		
	Baseline	PA Feedback	PA Feedback with JITAI	Baseline		PA Feedback	PA Feedback with JITAI
1	2.9 (0.9)	3.0 (0.0)	0.0 (0.0)	11.0 (2.8)		9.0 (0.0)	9.0 (0.0)
3	25.1 (11.8)	26.0 (2.3)	28.2 (1.3)	22.6 (6.2)		19.8 (11.8)	11.0 (1.2)
4	7.0 (5.7)	9.0 (2.5)	12.1 (1.0)	11.8 (4.2)		9.0 (0.0)	9.0 (0.0)
5	0.0 (0.0)	0.0 (0.0)	0.0 (0.0)	9.0 (0.0)		9.0 (0.0)	9.0 (0.0)
6	22.9 (15.1)	21.8 (25.5)	27.2 (38.5)	24.6 (14.3)		29.8 (3.3)	30.5 (3.5)
7	16.9 (9.7)	27.0 (7.6)	25.8 (1.7)	38.1 (7.9)		30.3 (4.9)	33.5 (7.6)
8	27.0 (33.2)	78.7 (18.5)	49.0 (2.8)	26.7 (12.4)		20.3 (2.1)	21.0 (2.8)
9	20.5 (41.0)	41.7 (36.1)	35.3 (34.5)	40.5 (6.2)		45.7 (7.6)	37.7 (5.4)
10	16.5 (1.5)	20.4 (2.4)	18.7 (2.5)	25.7 (4.6)		23.7 (2.9)	24.8 (2.9)
12	33.0 (7.5)	31.7 (5.5)	27.4 (3.9)	25.5 (1.7)		22.3 (1.5)	20.0 (4.7)
13	24.9 (5.3)	28.8 (4.5)	17.0 (4.1)	27.0 (9.9)		35.0 (4.7)	31.8 (3.9)
15	16.4 (12.9)	19.1 (3.2)	18.4 (0.8)	29.5 (3.7)		22.6 (4.4)	30.2 (8.2)
16	19.7 (6.5)	14.7 (0.6)	18.3 (0.5)	19.5 (5.5)		14.0 (2.9)	21.5 (0.7)
18	57.8 (15.8)	55.7 (5.9)	57.8 (9.6)	40.4 (4.3)		42.0 (2.6)	42.3 (2.1)
19	48.6 (22.6)	48.0 (3.4)	51.5 (9.5)	41.3 (6.7)		29.8 (5.6)	26.6 (2.3)
20	36.5 (14.8)	48.7 (6.2)	69.0 (12.6)	50.2 (7.6)		57.6 (1.1)	54.8 (2.3)
Group	23.5 (15.3)	29.6 (20.7)	28.5 (19.7)	27.7 (11.9)		26.2 (13.9)	25.8 (13.0)

### PA levels

Individuals received feedback on PA level, in terms of EE and distance traveled, on their overall PA level in the second and third phases of the study. Table 3 shows the PA levels for 16 individuals who completed the study. The EE, light-intensity PA, and moderate-intensity PA varied across phases within each participant and across participants over the study. The light-intensity PA duration over the 24-hour period for a day (or 1440 min) is higher than self-reported leisure time PA using the LTPAQ-SCI (Table 1) because it includes any PA that are not moderate- or vigorous-intensity PA. The moderate-intensity PA detected by the system is higher than the self-reported leisure time PA because it includes moderate-to-vigorous PA throughout the day. Table 4 shows within-participant PA level comparison for PA Feedback and PA Feedback with JITAI compared to baseline. Previous research by Bond *et al*. showed that a smartphone-based intervention led to a significant within-participant increase in moderate PA levels (5 to 7%) for adults who were overweight/obese [30]. Based on Bond *et al*.*’s* research [30], we chose a 10% of change in PA level as a “considerable” amount for participants in our study. Within-participant comparisons indicated that only two of the 16 participants who completed the study had considerably higher EE (>10%; participant ID#: 10) or lower EE (<-10%; participant ID#: 7) during PA Feedback with JITAI compared to the baseline. The results indicated that participants were able to considerably increase (>+10%) their light- and/or moderate-intensity PA during PA Feedback with JITAI (light-intensity: n = 6; moderate-intensity: n = 9) phase. In contrast, a smaller number of participants had a considerable decrease (<-10%) in their light- and/or moderate-intensity PA during PA Feedback with JITAI (light-intensity: n = 3; moderate-intensity: n = 7) phase. Furthermore, compared to the PA Feedback with JITAI phase a smaller number of participants were able to considerably increase their light- and/or moderate-intensity PA during the PA Feedback (light-intensity: n = 4; moderate-intensity: n = 3) phase. While most of the participants indicated that they were performing a higher level of light- and/or moderate-intensity PA during the PA Feedback and PA Feedback with JITAI phases, few participants indicated that chronic pain (participant ID#: 6, 3, 8), being busy at work (participant ID#: 6), weather (participant ID#: 6, 7, 8), hospitalization not related to the study (participant ID#: 7, 19, 20), and lack of accessible resources (participant ID#: 15) led to a decrease in PA levels.

**Table 3:** Mean (SD) PA levels across each phase of the study in the community, as measured by the system.

ID#	EE in kcal			Light-intensity PA in min			Moderate-intensity PA in min		
	Baseline	PA Feedback	PA Feedback with JITAI	Baseline	PA Feedback	PA Feedback with JITAI	Baseline	PA Feedback	PA Feedback with JITAI
1	2135.2 (240.2)	2105.0 (118.1)	2269.1 (127.1)	974.3 (261.0)	915.6 (343.2)	1112.7 (179.3)	36.3 (16.8)	32.6 (11.3)	54.9 (21.9)
3	2425.4 (303.3)	2391.1 (160.2)	2584.6 (206.7)	992.6 (315.6)	1007.4 (341.2)	1147.9 (241.5)	52.1 (19.7)	50.3 (27.7)	70.4 (31.5)
4	2172.9 (245.7)	2221.5 (190.1)	2224.8 (124.3)	1053.6 (266.5)	1100.8 (215.9)	1129.0 (305.8)	83.3 (28.8)	90.7 (28.4)	110.8 (36.3)
5	2409.5 (375.7)	2429.9 (234.0)	2395.5 (182.3)	903.9 (420.3)	1243.9 (96.7)	1123.8 (248.0)	101.8 (31.8)	128.1 (47.7)	124.5 (32.2)
6	2573.7 (256.5)	2641.8 (178.0)	2330.1 (263.3)	1022.2 (289.4)	1235.6 (189.4)	1245.3 (218.6)	129.6 (35.5)	107.6 (37.9)	62.4 (31.5)
7	2176.5 (224.8)	2022.3 (240.7)	1848.1 (158.6)	1139.4 (196.9)	1227.4 (187.3)	1142.8 (305.0)	96.2 (42.0)	64.9 (41.5)	39.8 (31.5)
8	2646.1 (275.4)	2607.1 (192.5)	2607.1 (141.8)	1092.5 (299.6)	1214.8 (178.7)	1314.9 (128.5)	83.4 (31.1)	68.7 (32.5)	65.0 (22.8)
9	1540.2 (195.9)	1518.5 (54.7)	1517.1 (56.0)	1284.5 (192.3)	1209.1 (298.1)	1142.7 (317.6)	40.0 (15.3)	35.6 (8.2)	31.9 (13.0)
10	1285.5 (167.2)	1363.2 (125.9)	1420.4 (145.4)	1073.8 (186.3)	1057.0 (248.5)	910.5 (302.2)	50.0 (43.0)	70.6 (40.1)	89.0 (39.7)
12	1941.0 (287.0)	1958.9 (104.7)	1925.5 (129.7)	1153.6 (276.6)	1133.6 (260.4)	1143.3 (263.3)	42.6 (19.6)	49.7 (20.6)	48.2 (24.4)
13	2321.3 (277.6)	2302.3 (77.1)	2335.6 (89.9)	1005.1 (313.6)	864.5 (259.5)	993.2 (361.6)	29.9 (16.6)	28.3 (13.0)	42.8 (13.7)
15	2515.0 (167.4)	2473.6 (193.0)	2283.2 (156.9)	1142.5 (189.0)	1158.0 (222.8)	1165.0 (273.9)	63.4 (25.9)	62.7 (25.4)	42.8 (20.9)
16	1833.0 (304.3)	1834.0 (114.2)	1867.3 (63.3)	971.8 (320.1)	831.5 (224.8)	781.1 (303.3)	34.6 (18.9)	34.9 (22.8)	34.0 (16.1)
18	2619.4 (176.0)	2628.1 (243.1)	2685.8 (185.7)	1221.2 (193.4)	1197.9 (111.9)	1183.9 (241.1)	103.7 (30.3)	110.6 (40.1)	115.8 (29.3)
19	1850.8 (281.6)	1788.7 (184.9)	1932.8 (218.0)	1281.4 (143.3)	1225.2 (235.9)	1294.9 (175.0)	68.6 (34.3)	60.2 (27.6)	53.1 (26.0)
20	1288.9 (205.7)	1270.1 (74.2)	1312.5 (81.4)	1222.0 (237.8)	1261.4 (223.5)	1193.5 (260.7)	45.9 (21.2)	44.7 (31.2)	52.8 (33.3)
Group	2108.4 (448.7)	2097.3 (445.8)	2096.2 (424.8)	1095.9 (116.2)	1117.7 (142.2)	1126.5 (134.8)	66.3 (30.0)	65.0 (30.2)	64.9 (29.6)

**Table 4:** Within-participant % change (SD) in PA level for PA Feedback and PA Feedback with JITAI compared to baseline.

ID#	EE		Light-intensity PA		Moderate-intensity PA	
	PA Feedback	PA Feedback with JITAI	PA Feedback	PA Feedback with JITAI	PA Feedback	PA Feedback with JITAI
1	-1.3 (5.4)	5.7 (5.5)	-5.2 (33.1)	16.8 (26.2)	1.7 (36.7)	61.3 (48.2)
3	-1.4 (6.7)	6.3 (8.5)	0.3 (34.5)	15.3 (20.4)	-13.9 (39.0)	24.5 (46.7)
4	2.2 (8.7)	2.4 (5.7)	3.2 (22.3)	10.1 (9.2)	3.3 (29.5)	19.2 (35.8)
5	1.7 (10.6)	1.7 (7.6)	40.2 (6.9)	29.3 (20.7)	25.8 (46.8)	22.3 (31.6)
6	1.8 (8.5)	-9.3 (7.9)	25.6 (7.2)	26.5 (14.3)	-11.6 (32.2)	-51.8 (22.4)
7	-7.1 (11.1)	-15.1 (7.3)	25.6 (17.2)	9.0 (22.0)	-8.4 (66.6)	-61.2 (17.8)
8	-1.6 (7.1)	-1.8 (5.2)	15.9 (15.2)	28.3 (14.9)	-15.7 (38.5)	-20.5 (27.3)
9	-1.3 (3.3)	-1.1 (3.5)	0.6 (6.6)	-13.0 (24.2)	-2.5 (20.4)	-12.1 (35.1)
10	6.0 (9.8)	10.5 (11.3)	2.6 (18.5)	-15.2 (28.1)	34.8 (74.7)	78.0 (79.5)
12	0.9 (6.1)	-0.9 (6.1)	0.2 (18.6)	3.6 (23.7)	28.5 (52.8)	11.0 (49.5)
13	-0.8 (3.3)	0.6 (3.9)	-20.1 (20.9)	-4.5 (34.1)	-20.1 (35.3)	24.2 (40.1)
15	-0.7 (7.5)	-9.1 (6.5)	1.5 (12.4)	0.4 (20.4)	4.2 (45.3)	-29.9 (29.5)
16	1.1 (6.4)	1.4 (3.5)	-16.5 (20.4)	-24.0 (32.0)	-8.5 (60.7)	-12.3 (37.2)
18	1.1 (9.5)	3.5 (7.6)	-4.0 (7.6)	-0.3 (6.9)	-0.7 (27.0)	11.0 (20.7)
19	-3.4 (10.0)	4.7 (11.9)	-3.7 (10.9)	0.3 (9.2)	-12.6 (40.1)	-22.0 (38.5)
20	0.4 (7.1)	3.7 (6.8)	0.9 (5.6)	-4.2 (14.5)	-17.8 (57.4)	13.9 (70.1)

### Use of JITAI

Exit interviews indicated that all participants found JITAI easy to use. Most of the participants reported that they did not have any issues wearing the smartwatch for the study. Two of the participants indicated connection issues between the smartwatch and smartphone. Similarly, many of the participants had minor to no issues using the wheel rotation monitor on their wheelchair. Six participants indicated that they had issues with observing a change in the distance traveled when they had switched the wheelchair wheel, which had the wheel rotation monitor’s magnet attached to it. The wheelchair wheel got switched when participants were unloading their wheelchair from a car or in other situations that required the wheels to be removed. Participants indicated that over time they became more aware that the wheel needed to be placed on the correct side of the wheelchair axel for the distance parameter to work. Thirteen of the participants indicated that they had no issues using the app, once it was set up. Three of the participants indicated that it took them between one to three days to get used to the PHIRE app. Three of the participants indicated that they sometimes had issues with the PHIRE app as it related to connectivity with the other devices or the app freezing when an EMA popped up. Participants indicated that they were able to resolve any major technological issues by contacting the study team. Most of the participants indicated that the JITAI system did not disrupt their everyday activities. One participant indicated that the EMA notifications interrupted their activity. The other participant was concerned that the audio notifications associated with the EMA may disturb his family members. Quantitative analysis indicated that the participants completed 75% (median) of the EMAs that were delivered to them over the course of the study.

Participants differed in their reactions to the system’s PA feedback and JITAI notifications during the PA Feedback and PA Feedback with JITAI phases. While five participants expressed that they thought the PA feedback during PA Feedback phase was generally useful, three participants felt it was not useful. On the contrary, nine participants indicated that they found the JITAI notifications during the PA Feedback with JITAI phase useful. And five participants expressed that the JITAI notifications were not useful to them. Participants expressed desires to have more control over the prompting, which activities could be tracked, and all four aspects of the active notification (i.e., timing, content, intensity, and frequency). Eleven of the sixteen participants indicated that they were willing to continue participation in the study if it was extended. All sixteen participants indicated that they would recommend this study and the PHIRE app to their friends.

## Discussion

Lack of regular PA is a major concern among individuals with SCI who are at an elevated risk of mortality due to cardiovascular diseases, diabetes, and lung disease [4]. Regular PA and exercise interventions have been linked with improved outcomes and healthier lifestyles among individuals with SCI [3]. To address this need, we have developed a sensor-based JITAI to measure and motivate PA in real-time. The JITAI uses machine learning algorithms to detect wheelchair-based PAs [10] in real-time, every minute, and then uses information about bouts of PA to provide a JITAI intervention to individuals with SCI as they go about their everyday lives. Compared to the other research studies that have validated activity monitors to track PA levels in individuals with SCI [8–10], our study extends the state-of-the-art research by providing real-time PA intervention based on a person’s actual PA behavior in the community.

### PA levels

The information provided during the PA Feedback with JITAI phase assisted majority of individuals with SCI (69.0%) in considerably increasing (>10.0%) their light- and/or moderate-intensity PA. Self-awareness of PA levels, possibly heightened by automatically-delivered feedback, has the potential to increase individuals’ PA levels [31]. However, PA information alone may not motivate individuals to substantially increase their regular PA levels [31]. Exit interviews at the end of the study indicated that participants felt motivated when the JITAI provided them with context-sensitive feedback and encouragement. These JITAI-based PA interventions provided positive reinforcement to the participants allowing them to continually perform small increments of moderate-intensity PA, rather than just reminding them about a broad general goal independent of their actual behavior. Furthermore, the feedback about the minutes of moderate-intensity PA accomplished for the day compared to their previous day (Fig 3D) enhanced participants’ engagement with the JITAI. Future research should identify additional personalized decision rules for the JITAI that might increase an individual’s engagement with the system and motivate an individual to perform more PA, thus leading to a healthier lifestyle [3, 5]. Results of this study are in agreement with Klasnja *et al*. who evaluated a JITAI in increasing steps of sedentary individuals from the general population [14]; the study showed that over the course of 6-weeks, the participants (n = 37) increased their average step count by 24% [14].

While majority of the participants indicated performing a higher level of PA during the PA Feedback and PA Feedback with JITAI phases of the study, a minority of the participants indicated that aspects such as chronic pain, being employed, weather, hospitalization, and lack of accessible resources led to a decrease in PA levels. Seven of 16 participants who completed the study were hospitalized due to various health conditions not related to our study (highlighting the challenges of conducting pilot studies with this community). Future studies should take the high rate of hospitalizations among individuals with SCI into consideration prior to conducting a longitudinal PA intervention. In addition, since EMAs were used to assess the type of PA a person was performing in the community, they could have contributed to reactivity and thus must be considered to be part of the intervention. However, most of the participants indicated that the JITAI system did not disrupt their daily activities. A key highlight of this study was that all sixteen participants who completed the study indicated that they would recommend the JITAI system to their friends to track and improve their PA levels in the community.

While results from this study cannot be directly compared with prior research, they are consistent with other studies [17, 18] that demonstrated an increase in PA levels of individuals with SCI due to behavioral interventions and delivery of PA guidelines [3]. Nooijen *et al*. evaluated whether behavioral interventions delivered during rehabilitation lead to a more active lifestyle [17]. PA was assessed with three body-worn accelerometers over a duration of one to four days at discharge, and at 6- and 12- months post-discharge from an inpatient rehabilitation facility. The study indicated that the intervention group (n = 11), which received 13 individual sessions delivered by a coach trained in motivational interviewing, had significantly higher overall wheeled PA at 12 months (25 min/day; 95% CI: 1 to 50) than the control group (n = 11) [17]. Similarly, the results of our study showed that the sensor-enabled JITAI has the potential to support individuals with SCI on a day-to-day or moment-to-moment basis, as they try to improve their PA levels.

In another study, Pelletier *et al*. conducted a 16-week randomized controlled trial to evaluate the effectiveness of the PA guidelines for adults with SCI in improving their physical fitness [18]. Individuals with SCI in the PA guideline group (n = 11) significantly improved their peak aerobic capacity (relative volume of oxygen (VO_2peak_): 17.2%, absolute VO_2peak_: 9.9%), submaximal power output (26.3%) and strength (range: 11.5–38.9%) compared to the control group (n = 8) [18]. Our JITAI was used to improve moderate-intensity PA, a recommendation by the clinical PA guidelines for individuals with SCI [32]. Future studies should evaluate JITAI-based technologies that incorporate both aerobic and strength-based PA to improve the health and quality of life of individuals with SCI.

### Study limitations

A sample size of 20 participants with SCI is small and the PA Feedback with JITAI was tested for only one month; thus, conclusions about the effectiveness of these pilot results should be interpreted cautiously. Non-randomization of the second (PA Feedback) and third phases (PA Feedback with JITAI) of the study may have introduced order effects. Limited statistical analyses were conducted due to the small sample size and non-randomization of the participants to the second or third phases of this study. Future research should implement a micro-randomized trial study design to assess the effect of JITAI on participants’ PA levels over time. A micro-randomized trial design allows treatments to be sequentially randomized throughout the study, which enables causal modeling of proximal effects of the intervention components and assessment of time-varying moderation of those effects [14, 33, 34]. The use of multiple real-time wireless sensors sometimes led to temporary data loss when participants: i) accidentally switched off the Bluetooth on the smartphones or placed the smartphones in airplane mode, and ii) connected their smartphone to multiple Bluetooth devices.

## Conclusions

To our knowledge, this is the first study to test a sensor-based, real-time, JITAI that passively measures PA levels and provides tailored feedback in response to individuals with SCI. The results of this pilot study indicate that such a system has potential to improve light- and/or moderate-intensity PA of individuals with SCI.

## Supporting information

S1 File Study protocol: The Institutional Review Board approved study protocol. (PDF)

S2 File Demographics and basic information questionnaire: Participants provided answered survey related to demographics, SCI, wheelchair information, health and PA history, smartphone use and fatigue. (PDF)

S3 File TREND Statement Checklist: Transparent Reporting of Evaluations with Nonrandomized Designs checklist. (PDF)
