# Correction: Mobile health-based physical activity intervention for individuals with spinal cord injury in the community: A pilot study

**DOI:** 10.1371/journal.pone.0225490

**Published:** 2019-11-14

**Authors:** Shivayogi V. Hiremath, Amir Mohammad Amiri, Binod Thapa-Chhetry, Gretchen Snethen, Mary Schmidt-Read, Marlyn Ramos-Lamboy, Donna L. Coffman, Stephen S. Intille

## Notice of republication

Incorrect versions of Figs 2 and 3 were published in error. This article was republished on November 7, 2019 to correct for this error. Please download this article again to view the correct version.
