# Supplementary material for: Mobile health-based physical activity intervention for individuals with spinal cord injury in the community: A pilot study
Source: PLoS One. 2019 Oct 15;14(10):e0223762. doi: 10.1371/journal.pone.0223762 (PMC6793862; doi:10.1371/journal.pone.0223762)
Supplement: S1 File — (PDF) [file pone.0223762.s001.pdf]

1) Abstract of the study

Lack of regular physical activity (PA) in the general population is a top public health concern, and this problem is even more acute among individuals with spinal cord injury (SCI). Individuals with SCI also experience secondary conditions such as pain, fatigue, weight gain, and deconditioning many of which are considered preventable through PA and exercise interventions. To address this need, we have developed and validated a smartphone based Physical Activity Monitor System (PAMS), which consists of a smartphone, a smartwatch and a wheel rotation monitor. PAMS utilizes statistical machine learning algorithms to detect various wheelchair-based PAs in community settings, estimate energy expenditure, and provide real-time feedback about PA levels to individuals with SCI.

The objective of this study is to develop algorithms that incorporate each individual’s automatically detected PA level and a clinician’s PA level recommendation to provide a smartphone application that helps a person set safe (23) and highly personalized PA goals. By adapting the goals in real-time based on the person's actual behavior, the system aims to keep the individual feeling positive and motivated towards a change in the PA behavior.

Aim 1: Extend and utilize PAMS to track PA levels, sedentary behavior, and secondary conditions such as pain (24), fatigue (25), and deconditioning (26) in community settings.

Aim 2: Extend and utilize PAMS to passively monitor PA and provide continuous, but passive feedback about PA levels to individuals with SCI in community settings.

Aim 3: Extend and utilize PAMS to passively monitor PA and provide just-in-time persuasive and adaptive feedback to motivate individuals with SCI in community settings.

2) Protocol Title

Just-in-time adaptive feedback systems to assist individuals with SCI

3) Investigator

Shivayogi V. Hiremath, Ph.D.
Assistant Professor, Department of Physical Therapy
College of Public Health (CPH)
Pearson Hall 40, 1800 N Broad Street, Philadelphia, PA
19121Shiv.Hiremath@temple.edu, 215-204-0496

4) Objectives

The objective of this proposed study is to develop algorithms that incorporate each individual's PA level and a clinician's PA level recommendation to provide a smartphone application that helps a person set safe and highly personalized PA goals. By adapting the goals in real-time based on the person's actual behavior, the system aims to keep the individual feeling positive and motivated.

Aim 1: Extend and utilize PAMS to track PA levels, sedentary behavior, and secondary conditions such as pain (24), fatigue (25), and deconditioning (26) in community settings.

PA level is defined in terms of minutes (mins) of moderate or vigorous-intensity aerobic activity per week and number of days per week of muscle strengthening activities (2). PA guidelines for adults with disabilities recommend 150 mins of moderate or 75 mins of vigorous-intensity aerobic activity per week and, if possible, 2 days per week of muscle strengthening (2). We will expand the current PAMS smartphone application to allow for the user to provide ecological momentary assessments (EMAs) about pain (scores), fatigue (scores), and deconditioning (reduced capacity scores) on a regular basis.

Hypothesis 1a: PA level of individuals with SCI in community settings will be low (only 20% of the participants will be performing regular PA (4)) compared to the PA level recommended for individuals with disabilities in general.

Hypothesis 1b: Sedentary behavior, as measured by time duration of non-movement of individuals with SCI (as opposed to measured based on time seated in a wheelchair) will be high (75% of the participants will be sedentary for greater than 75% of daily waking hours (27)) compared to the general population.

Hypothesis 1c: Pain, fatigue and deconditioning measured over first month of the study will be negatively correlated (r= 0.3-0.5) with PA levels being performed.

Aim 2: Extend and utilize PAMS to passively monitor PA and provide continuous, but passive feedback about PA levels to individuals with SCI in community settings.

Self-awareness of PA levels has the potential to increase individuals' PA levels. However, PA information alone may not motivate individuals to substantially increase their regular PA levels.

Hypothesis 2: PA levels and sedentary behavior patterns of the participants, when obtaining passive feedback about their PA levels, during the second month of the study will not be significantly different compared to Aim 1.

Aim 3: Extend and utilize PAMS to passively monitor PA and provide just-in-time persuasive and adaptive feedback to motivate individuals with SCI in community settings.

Providing personalized messages at specific times and contexts when activity is actually being performed and proactively congratulating the participants when they achieve their goals will enhance system engagement and thereby lead to

behavior change (28). The adaptive feedback will be based on algorithms that take into account: i) each individual's current and previous PA levels and sedentary behaviors, ii) the clinician's PA level recommendations for that individual, iii) the context of the PA, and iv) the optimal level of PA that the individual can perform without increasing the risk of secondary conditions.

Hypothesis 3a: PA levels of the participants will be significantly higher for the third month of the study (light-level PAs by 25% and moderate-level PAs by 7% (27)) compared to their own baseline PA levels measured in Aim 1.

Hypothesis 3b: The sedentary behavior of the participants during the third month of the study will be significantly lower (time by 6% (27)) compared to Aim 1.

Hypothesis 3c: Pain, fatigue, and deconditioning during the third month of the study will be significantly lower compared to Aim 1.

5) Rationale and Significance

Rationale

The proposed work aims to build a just-in-time persuasive and adaptive feedback system that could help bridge the gap between clinical recommendations of PA level (to attain) and the regular PA level performed by individuals with SCI in the community. This adaptive feedback system can allow individuals with SCI, clinicians, and researchers to better monitor the health and PA levels of people in the community. The passive and just-in-time system will allow individuals to visualize the type of PAs they currently perform, the time they perform PA, and the context of their PAs. The information obtained from the adaptive feedback system will empower users towards increasing their PA levels in small increments by performing the activities that they prefer, rather than offering them a large general goal that they need to attain as per the PA guidelines. Another benefit of this adaptive feedback system is that it can provide PA suggestions that are safe for each participant based on a clinician's recommendation tailored to that individual.

The study will assess both passive real-time feedback and just-in-time persuasive and adaptive feedback provided through smartphones. The passive real-time feedback has the potential to make the individual self-aware of their PA levels. This approach may be effective in a small percentage of individuals who are very motivated to increase their PA levels. However, market analysis of activity monitors' adoption in the general population suggests that providing passive PA information may only lead to a short term adoption of using these products by many people, i.e. several weeks (65, 66). To achieve long-term engagement, which will be required to support long-term behavior change and maintenance (as well as new research), automatic detection of behavior may permit delivery of highly-tailored positive reinforcement and adaptive goal setting. The adaptive feedback will be based on the individual's current PA levels and

sedentary behaviors, the clinician's PA level recommendations for that individual, and the context of the detected PAs over prior days.

Significance

The use of just-in-time persuasive and adaptive feedback systems could have a large impact on health, PA level, reduced secondary conditions and improved quality of life for individuals with SCI. Based on the current literature and our previous studies, we find that there are a number of studies that developed and validated activity monitors in individuals with SCI both in laboratories and under free-living conditions. However, none of these tools are currently available for clinicians and individuals themselves to assist and change PA levels in the community. In addition, there is no published data about the patterns of PA and sedentary behavior of individuals with SCI in their homes and communities. This project will extend the state of the art in monitoring individuals with SCI to support additional research on just-in-time persuasive and adaptive feedback to increase PA levels of people with SCI, leading to recommendations for a healthier lifestyle (3, 5, 37). This pilot research could eventually lead to systems that support individuals on a day-to-day or moment-to-moment basis, as they try to increase physical activity by incorporating clinician recommendations into their everyday lives. The tool we will develop and validate will support providing adaptive feedback to individuals, based on passive recording of PA patterns and occasional self-report of feelings of pain, fatigue, and deconditioning. The tool will operate as people go about their everyday lives in their home and community environments. Researchers can use this type of PA monitor system to study and evaluate repetitive strain injuries related to wheelchair use (67, 68), effectiveness of exercise-based intervention programs at improving health and function (32, 51), psychosocial aspects and quality of life (7, 10, 51, 69), and interventions that deliver novel, tailored, timely, context-sensitive feedback based on a person's actual PA. Developing such a monitor system has the potential to allow evidence-based practice in wheelchair usage and prescription (70, 71).

Preliminary Data:

Dr. Hiremath's prior research involved developing a gyroscope based wheel rotation monitor (16) and combining this device with Dr. Intille's wearable accelerometer (60) to estimate PA levels in manual wheelchair users. An Android-based smartphone application was developed to receive data from both devices wirelessly to estimate PA in laboratory and community settings (20). We have developed and validated classification algorithms that detect wheelchair based PAs (21), and EE estimation models that quantify PA (22). In addition, Dr. Hiremath (20) and other researchers (63) at the Human Engineering Research Laboratories, University of Pittsburgh have performed usability testing of PAMS in both laboratory and community settings.

Development of gyroscope based wheel rotation monitor

We have developed and evaluated a wireless gyroscope-based wheel rotation monitor (G-WRM) that can estimate speeds and distances traveled by wheelchair users during regular wheelchair propulsion as well as wheelchair sports such as handcycling (16). G-WRM (Figure 1) can provide users with real-time feedback through a smartphone application. The percentage of errors for the angular velocities, speeds, and distances obtained from three prototype G-WRMs were less than 3% for all the test trials. The wireless data transmission tests indicated less than 0.3% of data loss. The results of this study indicated that the G-WRM is an appropriate tool for tracking and providing real-time feedback about wheelchair-related activities. PAMS with an additional battery pack can collect wheel rotation data for about a month without recharging.

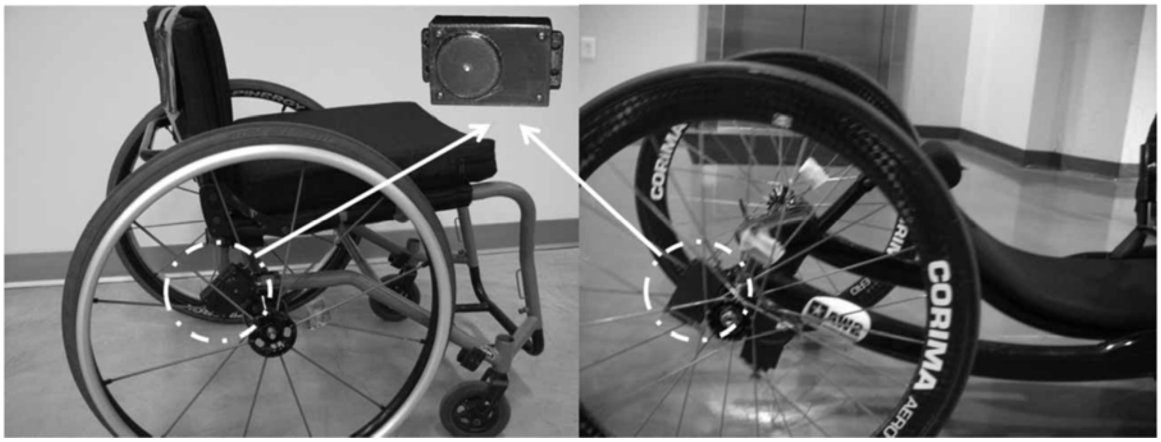

Figure 1: G-WRM secured to the spokes of a manual wheelchair and a handcycle.

Estimation of PA level using PAMS

Following the development of G-WRM we evaluated the performance of a PAMS to estimate the EE of wheelchair-based PAs in 45 manual wheelchair users with SCI. The system consists of a G-WRM (20) for capturing wheelchair movement and a wearable accelerometer (60) for tracking upper arm or wrist movements. The study developed and validated activity-specific EE estimation models for manual wheelchair users based on either the G-WRM and arm accelerometer (PAMS-Arm) or the G-WRM and wrist accelerometer (PAMS-Wrist). The criterion EE was measured using a portable metabolic cart. The overall EE estimation performance analysis was a two-step process (17) of first applying the classification algorithms, which detect the wheelchair-based PAs, and then applying the activity-specific EE estimation model.

Classification algorithms such as support vector machines and decision trees utilized sensor data from PAMS-Arm and PAMS-Wrist to detect and classify wheelchair-based PAs (21). An in-depth classification performance analysis of the best classifiers indicated that PAMS-Arm and PAMS-Wrist had similar overall classification accuracy (89.3% vs. 88.5%), precision (lower precision for resting and activities that may involve small wheelchair movements) and recall (lower

recall for resting and basketball). The analysis revealed that multimodal sensor information from PAMS-Arm and PAMS-Wrist had higher accuracy than the individual devices (G-WRM and the wearable accelerometer). The confusion matrix analysis for PAMS indicated that 89.3% of the PAs were correctly predicted. Further, the analysis indicated that misclassified PAs (non-diagonal) were classified into biomechanically similar PAs; for example, resting was classified as other household PAs in the stationary category, and basketball was labeled as a PA that involved wheelchair movement because of the intermittent wheelchair movement. Even though misclassification resulted in lower classification accuracies, grouping activities into similar type of wheelchair-based activities can lead to an estimate of the EE or metabolic equivalent of task values similar to the values obtained for correctly identified activities.

Seven activity-specific EE prediction models were successfully developed for PAMS-Arm and PAMS-Wrist (22). The EE estimation models chose demographic features such as weight of the person, lean body mass, height, and gender. This is not surprising considering that the total EE during resting and PAs for an individual depends on these parameters (64). The validation errors obtained from using the testing dataset, not used for developing models, ranged from overestimation to underestimation (positive percentages) for PAMS-Arm (mean signed error range: -16.2% to 29.8%; overall: -1.0%) and PAMS-Wrist (-12.6% to 22.4%; overall: -0.8%). The EE estimation error obtained by sequentially applying classification algorithms (21) and the estimation models showed that the overall EE error based on mean signed error was lower than 10% for both PAMS-Arm (-9.8%) and PAMS-Wrist (-5.7%). The mean signed error is commonly used in sports sciences as an overall performance indicator for PA monitors over a period of time (17). The low mean signed error indicates that both PAMS-Arm and PAMS-Wrist can estimate EE with low bias (<10%) in MWUs with SCI. Furthermore, the EE estimated for PAMS-Arm (Intraclass Correlation Coefficient – ICC(3,1) = 0.82, $p < 0.05$) and PAMS-Wrist (ICC(3,1) = 0.89, $p<0.05$) had moderate to high ICC values for the majority of PAs, thus indicating that the EE values estimated by PAMS-Arm and PAMS-Wrist are consistent with the EE measured by the portable metabolic cart. Overall, the validation analyses indicated that both PAMS-Arm and PAMS-Wrist can estimate EE with reasonable accuracy (<10% MSE) for MWUs with SCI in laboratory and community settings. The similar EE estimation performance by PAMS-Arm and PAM-Wrist suggest that wheelchair users can choose to wear the accelerometer on the wrist or the upper arm based on comfort.

Usability testing of PAMS in laboratory and community

Six manual wheelchair users with SCI evaluated PAMS in a laboratory setting (20). The overall score for the System Usability Scale was a mean of 94 ± 7, with 100 being the highest, thus indicating that PAMS has high usability and learnability. Furthermore, the median responses of the participants to the modified Technology Acceptance Model ranged from 4.5 to 7, with 7 being the highest, showing that PAMS was perceived as useful. All participants indicated that the information provided by the PAMS smartphone application about

distance travelled, speed, calories expended, and duration of moderate to high intensity PA, was specific and appropriate. The field-based usability study evaluated PAMS in the homes of 10 individuals with SCI for a week to identify problems encountered by users and to assess each user's experience and satisfaction (63). The average score on the System Usability Scale was 87 ± 13. All participants reported that they could envision themselves using PAMS to track PA levels on a regular basis and would recommend PAMS to other individuals who use wheelchairs.

Impact to the Field of Rehabilitation Sciences

The project is designed to improve our understanding of PA levels, sedentary behaviors and secondary conditions of individuals with SCI in their homes and communities. Testing of innovative strategies in personal health informatics through use of wearable monitors and mobile health technology will allow us to advance SCI research and preventive measures for chronic SCI. Individuals with SCI are stable post rehabilitation, but they are at higher risk for developing secondary conditions due to physiological changes and the use of assistive technology such as wheelchairs, which may further lead to sedentary behaviors. Accurate, real-time measurement of PA, where data are immediately available to apps on a smartphone, will enable not only more research on existing behavior and relationships to health outcomes, but also new research on innovative just-in-time persuasive and adaptive feedback systems that may help individuals with SCI become more active in everyday life (thereby improving their quality of life). Real-time, tailored feedback based on automatically-detected behavior is a new approach that could play an important role in future inpatient and outpatient rehabilitation interventions, but new tools are needed to make such research possible.

6) Resources and Setting

Dr. Hiremath has worked with more than one hundred individuals with SCI during his doctoral and postdoctoral training. Dr. Hiremath has established research history involving individuals with SCI, including relationship with healthcare providers and community/support groups.

Dr. Hiremath has built advanced algorithms to estimate physical activity in individuals with SCI. Dr. Hiremath has started a collaboration with Dr. Marlyn Ramos-Lamboy at MossRehab and Dr. Mary Schmidt-Read at Magee Rehabilitaion. Dr. Hiremath will be able to utilize this network to recruit participants for this study. All other staff involved in the study will be closely supervised by the investigators to ensure the quality of their work and adequate orientation to study goals and procedures. At least one of the investigators will be present for all data collection, consent discussions, and any study-related interactions with subjects.

Laboratory Space

Dr. Hiremath has a furnished laboratory space (420 square feet) dedicated to this project. The space includes cubicles that ensure privacy during telephone interactions with participants. The space also includes Internet jacks, telephones, lockable file cabinets, and standard office equipment and supplies to facilitate research-related activities. The lab is equipped with a conference area, an LCD projector, and videoconferencing capability.

Computing

Temple University has wireless access and a state of the art Technology Center with easy access to the Internet for any project staff. Research faculty and staff have access to a HIPAA compliant Data Center providing secure data storage and disaster recovery services. All staff use project dedicated, password protected computers. TUcloud enables Temple University departments to host applications and/or store data using the infrastructure of Temple's state-of-the-art data center. High Performance Computing (HPC) offers two high performance computing Linux environments to provide university researchers sufficient computing power to do their intensive data analysis.

Office

The PI and postdoctoral fellow have been provided office space within the Department of Physical Therapy, College of Public Health. The Department also provides work stations for all its graduate students and post-doctoral trainees and over 1,000 square feet devoted to shared resources, including document storage, printers, copiers, scanners, and shared digital network.

Collaborator at Temple

Dr. Gretchen Snethen is an Assistant Professor at the Department of Rehabilitation Sciences. A core component of the mission of the department is to promote independence and engagement in personally meaningful activities among individuals with disabilities. The work of the department is driven by an ecological and strengths-based approach that facilitates an individual's empowerment to participate independently within his or her community. This type of environment provides intellectual community and a philosophical framework suitable for the development of individualized, interest-based interventions. Dr. Snethen supervises two, externally funded recreational therapists who focus on supporting individuals with disabilities to identify community-based, recreation interests and provide on-going supports to increase independent engagement. These individuals can provide additional feedback on identifying barriers of and facilitators to participation, connecting activity interests with accessible resources, and providing individualized supports. The Temple University Collaborative on Community Inclusion of Individuals with Psychiatric Disabilities, housed within the Department of Rehabilitation Sciences also maintains a list of

recreation and leisure activities available in the Philadelphia region and specific information about physical accessibility.

Dr. Amir Amiri is a postdoctoral research fellow under Dr. Shivayogi Hiremath at the Personal Health Informatics and Rehabilitation Engineering Laboratory, Department of Physical Therapy. Dr. Amiri received his Masters Degree in Biomedical Engineering and a Ph.D. in Computer Science. His research interests include pattern recognition, biomedical signal processing, and body sensor network. He is currently working on internet-of-things, sensors, data analysis, cloud computing and intelligent systems that could be used for physical activity monitoring. Dr. Amiri has previously worked with pediatric population and adults with autism.

Facilities and Other Resources at Moss Rehab (Marlyn Ramos-Lamboy, MD)

MossRehab is a member of the Einstein Healthcare Network, the largest independent medical center in the Philadelphia region. MossRehab can provide comprehensive treatment for all the needs of their consumers in a single location, with a team of doctors and therapists working together to create a customized plan for each individual. The MossRehab Spinal Cord Injury Program is structured to move hand-in-hand with people following traumatic and non-traumatic SCI. The program is designed to help those affected with SCI achieve independence and lead productive and rewarding lives. At MossRehab, every patient is treated on an individual basis. The program begins by introducing the use of assistive technology, when needed, to allow common daily activities to become familiar once again. Both living space and work space are modified when they need to be made more accessible and stress-free. Family members are trained to provide assistance if needed. MossRehab also has a very active recreational therapy program for individuals with disabilities.

Facilities and Other Resources at Magee Rehabilitation (Mary Schmidt Read, PT, DPT, MS)

Magee Rehabilitation Hospital, in affiliation with Thomas Jefferson University, is designated as one of the nation's 14 Model Spinal Cord Injury Centers by the National Institute on Disability, Independent Living, and Rehabilitation Research (NIDILRR) within the Administration for Community Living (ACL), Department of Health and Human Services (HHS), and is the only Model SCI Center in the Delaware Valley.

The center, which has treated more than 3,000 persons with spinal cord injury, provides for the multidisciplinary coordination of emergency and acute medical/surgical care, rehabilitation beginning at the onset of acute care, vocational-evaluation and training, and lifetime follow-up care for persons with

spinal cord injury. With over 50 percent of persons with spinal cord injury admitted within three days of injury, the Regional Spinal Cord Injury Center has demonstrated a mortality rate of 5 percent and has significantly reduced the severe secondary complications of traumatic spinal cord injury.

Recruitment of Subjects with SCI

A total of 20 individuals with SCI will take part in the study. To account for attrition and other issues related to longitudinal studies (our study duration is 3 months) we will screen and recruit 30 individuals with SCI.

The research team will work with Dr. Ramos-Lamboy, a collaborator from MossRehab, and Dr. Schmidt-Read, a collaborator from Magee, to recruit participants from the MossRehab and Magee Rehabilitation, respectively (Philadelphia region). Participants will also be recruited from the MossRehab's and Magee Rehabilitation's weekly support group and peer mentoring program offered to individuals with SCI. Working with Drs. Ramos-Lamboy and Schmidt-Read will allow us to recruit and retain the participants who may be interested in the study.

In addition, we may recruit participants via flyers and advertisements in print media (magazines, newspapers, newsletters) and web-based postings. Flyers may be posted in local rehabilitation facilities, outpatient facilities, and disability organizations. The advertisements will be posted on the Temple University Physical Therapy Department's website (https://cph.temple.edu/pt/research-centers-labs/current-projects) and other related web-sites. Interested participants will contact (phone or email) or meet with the investigator to discuss the project and ask questions. Participants who meet the inclusion criteria and are free to exclusionary factors will advance with the testing procedures.

Research study procedures

<u>Aim 1 (detailed information about study procedures are provided below in the section Study Procedures and Data Analysis)</u>

The first visit can be at the participant's home or at Temple University, based on their choice. If their health care provider is MossRehab or Magee Rehabilitation then their first visit can be at their health care provider's facility. The participants will provide the informed consent and will answer questions about demographics, SCI, wheelchair information, and health and activity history (local environment and access to gym and recreation centers). The total time for the first visit will not exceed one hour and 15 minutes. If Dr. Ramos-Lamboy is their care provider then he will recommend them to continue their normal physical activity pattern. Otherwise Dr. Hiremath will recommend them to continue their normal physical activity pattern. The participants will be taught how to use and maintain PAMS.

<u>Aims 2 and 3</u>

Participants will answer survey questionnaires similar to those from their first visit (either at their home or at Temple University, based on the participant's choice). If Dr. Ramos-Lamboy is their care provider then he will provide clinical recommendations on how much physical activity they should strive to obtain and how they might accomplish that for the second and third months of the testing. Otherwise Dr. Hiremath will provide the standard clinical recommendations on how much physical activity they should strive to obtain and how they might accomplish that for the second and third months of the testing. Participants will continue to use PAMS and provide regular EMAs.

Though the study is minimal risk, if a study subject should require medical attention while at their home, Temple University, MossRehab or Magee Rehabilitation the study staff will call 911 emergency medical services if necessary to transport subjects to receive medical attention.

Approval from Temple University's IRB and Biosafety, Moss Rehabilitation Research Institute's IRB, Magee Rehabilitation's IRB, Northeastern University's IRB will be obtained prior to commencing the research.

Facilities and Resources at Northeastern University (Stephen Intille, PhD)

The facilities and resources available to Dr. Intille at Northeastern University include what is required to successfully complete the proposed project. Dr. Intille is appointed in both the College of Computer and Information Sciences and the Bouvé College of Health Sciences, and he has access to resources provided by both Colleges.

<u>Laboratory</u>

The College of Computer and Information Science houses the Human-Computer Interaction (HCI) Laboratory, where mobile software development and pilot usability testing will take place. The HCI Laboratory is a ≈2,400 sq. ft. facility dedicated to support the development and evaluation of advanced human computer interface technologies, especially personal health informatics technologies. There is adequate laboratory space for the faculty and staff involved in the proposed project.

<u>Computer</u>

The HCI Laboratory has a range of computer workstations designed for advanced software development, especially with mobile devices. The lab includes a variety of mobile phones and smartwatches and equipment for mobile phone system programming. The College of Computer and Information Science has shared computer labs and wired and wireless networks that support research and teaching and meet the needs of the proposed work. Access to mathematical modeling and pattern recognition software such as Matlab is also available.

Dr. Intille's research group operates virtual machine research servers for projects. The primary research server used for mobile study data collection is a

firewalled virtual machine that is based on KVM and QEMU, and is hosted on an Ubuntu server that has 24 cores and 100G RAM. The host server is physically located in a secure datacenter in the College of Computer and Information Science (West Village H) at Northeastern University. The server VM image is stored on a separate hard drive from host server. The host server and the research VM are being monitored by a third-party system, Pingdom that provides alerts about server downtime. The server software uses a MySQL relational database, Apache Tomcat web server, and has additional components such as postfix and PHP. Custom software developed by Dr. Intille's research team is used for data uploading and visualization.

<u>Office</u>

The HCI laboratory includes faculty offices and graduate student desk space. There is adequate office space Dr. Intille and the graduate student involved in the proposed effort. Dr. Intille has a private office to store data in locked file cabinets.

PA tracking in the community

The data about movement (arm and wheelchair movement) and EMA data (without HIPAA identifiers) collected using the PAMS application will be directly uploaded to the secure virtual machine research servers at Dr. Intille's research groups. All precautions will be taken to keep this data secure. More information about PAMS application is presented in the Instrumentation section of Study design.

Mobility Tracking in the Community

The data about mobility collected through the global positioning system (GPS) sensor in the phone by the AccuTracking application will be sent to a secure online database at one minute intervals. GPS data collection can be monitored by logging into the AccuTracking website, and at the end of the tracking period, each subject's data will be downloaded to our HIPAA secure servers at Temple University (more information about this application is presented in the Instrumentation section of Study Design).

7) Prior Approvals

Approval from Temple University's IRB and Biosafety, Moss Rehabilitation Research Institute's IRB, Magee Rehabilitation's IRB, Northeastern University's IRB will be obtained prior to commencing the research.

8) Study Design

a) Recruitment Methods

<u>Number of Subjects</u>

A total of 20 individuals with SCI will take part in the study. To account for attrition and other issues related to longitudinal studies (our study duration

is 3 months) we will screen and recruit 30 individuals with SCI. The sample size for this pilot study is based on budget constraints and other pilot studies (14, 15, 18). This study will provide the pilot data required to compute the power of future studies.

Recruitment

The research team will work with Dr. Ramos-Lamboy, a collaborator from MossRehab and Dr. Schmidt-Read from Magee Rehabilitation to recruit participants (Philadelphia region). Participants will also be recruited from the MossRehab's and Magee Rehabiliation's weekly support group and peer mentoring program offered to individuals with SCI. Working with Drs. Ramos-Lamboy and Schmidt-Read will allow us to recruit and retain the participants who may be interested in the study.

In addition, we may recruit participants via flyers and advertisements in print media (magazines, newspapers, newsletters) and web-based postings. Flyers may be posted in local rehabilitation facilities, outpatient facilities, and disability organizations. The advertisements will be posted on the Temple University Physical Therapy Department's website (https://cph.temple.edu/pt/research-centers-labs/current-projects) and other related web-sites. Interested participants will contact (phone or email) or meet with the investigator to discuss the project and ask questions. Participants who meet the inclusion criteria and are free to exclusionary factors will advance with the testing procedures.

Payment

Subjects will be paid $15 for the enrollment interview and up to $20 per week for answering weekly surveys and daily short surveys on the smartphone for three months. Subjects will participate in study for three months (with a minimum of 28 days and a maximum of 31 days per month). Subjects will also be paid $45 for your final interview and return of the equipment. Subjects will be paid via gift cards after each month of the study. Subjects will be paid up to $40 to cover costs associated with parking or taking a public transport for their visits. In addition, if the subject chooses to use their own Android based smartphone to install the application (which will increase the compliance) and track physical activity then they will be reimbursed up to $45 for their data plan per month.

b) Inclusion and Exclusion Criteria

Participants will be included if they are 18-65 years of age, use a manual wheelchair as their primary means of mobility (80% of time), can self-propel their wheelchair, have a diagnosis of SCI, are medically stable, 6-months post injury, and have experience using a smartphone. Participants will be excluded if they have active pelvic or thigh wounds, have a history of cardiovascular disease or are pregnant (self-report). We will target our

recruitment effort towards both men and women with SCI, and people of diverse race and ethnicity.

c) Study Timelines

Participants will be enrolled in the study for three months. The anticipated duration to enroll all subjects is two years. Our hope is to complete the study by August, 2018.

d) Study Procedures and Data Analysis

Study Procedures

A total of 20 individuals with SCI will take part in the study. Participants will be included if they meet the inclusion criteria (discussed below in the Human Subjects section). Each individual will take part in the study for 3 months.

The first month will involve collecting baseline PA levels. The second and third months of the study will provide the participants with passive feedback (20, 21) and just-in-time persuasive feedback about their PA levels, respectively. Self-assessments of pain, fatigue, and deconditioning will be collected using EMA (i.e., questions prompted on the smart phone) throughout the study on a regular basis. Participants will be required to make three visits. All three visits can be at their home or at Temple University, based on their choice. If their health care provider is MossRehab or Magee Rehabilitation Hospital then their first visit can be at their health care provider's facility. At the start of each of the second and third months of the study, the investigators will either visit each participant's home or meet with the participant at the university-based laboratory, based on the participant's preference. The meeting will allow the investigators to program new models into the smartphone application and to train the participants how to use passive and persuasive feedback conditions.

<u>Instrumentation</u>

PAMS incorporates a G-WRM or a wheel-rotation sensor fixed to the wheelchair wheel and a wearable accelerometer device. The G-WRM is a self-enclosed rechargeable Bluetooth-based wireless device that contains six reed switches and a two-axis gyroscope to measure the angular velocity of the wheelchair wheel (16). The G-WRM will be placed in a quick release case secured to the spokes of the wheelchair wheel (20, 63). A wheel-rotation sensor is a bike sensor (PanoBike Speed sensor or equivalent (85)) that can measure speed of the wheel rotation. Both G-WRM and wheel-rotation sensors transmit information to an Android based smartphone via Bluetooth®.

The wearable accelerometer will be a Android smartwatch (model LG R or equivalent) that captures body motion using a tri-axial accelerometer and transmits information to an Android based smartphone. This system is being

used in ongoing data collection by Dr. Intille's research group. The smartwatch will be worn on the right wrist of the user. Sensor data collected from the accelerometer will include tri-axial acceleration. The G-WRM or wheel-rotation sensor and the accelerometer will be time synchronized by the Android smartphone that collects sensor data and provides real-time feedback to the user. The Android smartphone will be either secured to the participant's waist or placed in a pouch under the wheelchair seat, which is commonly used by individuals who use manual wheelchairs.

<u>Android application</u>

Currently, the PAMS application can receive wireless sensor information from G-WRM and a wearable wireless accelerometer to detect wheelchair-based PAs and estimate PA levels in the laboratory and community settings. The PAMS app (Figure 2) will be extended to have the ability to obtain self-reported assessments using EMA of pain (24), fatigue (25), and deconditioning levels (26). The PAMS app will also provide passive and just-in-time persuasive feedback about PA levels to individuals with SCI in community settings. In the passive feedback condition, the phone will display information about the current PA levels during each day of the week from the start of the study through the current day (as shown in Figure 2). The data displayed will be estimated using the PAMS system. The PA detection and estimation models use statistical measures such as time and frequency domain features, calculated based on the data collected from G-WRM and a wearable accelerometer. We will also include the participant's parameters such as weight, height, gender, age, injury characteristics, wheelchair weight and basal metabolic rates as some of these variables are substantial predictors of energy expenditure (EE) estimation for various activities.

Starting in month three, the persuasive feedback condition will provide personalized messages at specific times and contexts (e.g. after 10 mins of arm-ergometry or 15 mins of basketball), and congratulating the participants when they achieve their daily goals. Participants will also be congratulated if they perform regular PAs on a daily or weekly basis which allows them to sustain their current PA (44, 45, 62). When PA is detected, the app will ask participants to extend the activity by a small amount (e.g. 2 to 5 extra mins of arm-ergometry, if a person has been engaged in the activity for 20 mins). Questions prompted on the phone or smartwatch will be used to determine, in the moment, why or why not someone follows a recommendation.

In situations where there is data loss, due to one of the devices being not charged, or one of the devices not being worn/carried, data will be collected from the other devices and supplemented by self-report. Collecting this data will allow the participants to self-report the types of PAs later in the day, assisted by visualizations of data that were collected, as Dr. Intille has done in prior work (59). The system will send a brief report (few kilobytes) using the participant's smartphone to a remote server on a daily basis, indicating the size of data

collected and, if needed, any anomalies with the testing session. This will permit remote compliance checking and reduce the risk of data loss.

Mobility Tracking in the Community

AccuTracking (www.accutracking.com) software will be used for tracking the locations and movement of study participants. AccuTracking has been used by individuals, government agencies, businesses, and researchers in order to track the location of family members, vehicles, employees, or subjects in real time. The software has been used in several published studies (84) and is currently used by Dr. Snethen's research group at Temple Univesity. No action will be required from the individual being tracked other than carrying their smartphone or the study smartphone with them. AccuTracking automatically starts whenever the phone is turned on, and runs in the background. However, if the phone is turned off, tracking would stop. The participant can also disable the software by contacting the research study team at any time point. GPS tracking will happen whenever the individual is outdoors. The mobility tracking information will be sent to a secure online database at one minute intervals. GPS data collection can be monitored by logging into the AccuTracking website, and at the end of the tracking period, each subject's data will be downloaded to our HIPAA secure servers at Temple University.

EMAs

Self-report assessments of pain, fatigue, and deconditioning will be requested every week using EMA on the smartphone. All of these assessments will be provided in a single session on a fixed day and approximately the same time of the week depending on the participant's availability. The estimated time for completing these EMA surveys is between 10 and 12 mins based on us filling the surveys. The choice of one assessment per week is aimed at balancing the participant burden of providing EMAs and obtaining regular data.

The pain survey questionnaire will include the 7-item Chronic Pain Grade questionnaire, which assesses pain intensity and pain-related disability (72). The questionnaire has been validated with high internal consistency (72-75) and has been utilized to assess chronic pain associated with spinal cord injuries in the community (24). Characteristic pain intensity will be calculated by averaging 0 to 10 ratings of current pain, worst pain in the past 6 months, and average pain in the past 6 month (questions 1 to 3), and then multiplying the average by 10 (72). Pain-related disability scores will be calculated by averaging 0 to 10 ratings of pain interference with daily, social, and work/housework activities in the past 6 months (questions 5 to 7), and then multiplying the average by 10 (72). The Chronic Pain Grade questionnaire classifies individuals with pain into 4 categories: Grade I – low pain intensity and low pain-related disability; Grade II – high pain intensity and low pain-related disability; Grade III – moderate pain-related disability; and Grade IV – severe pain-related disability. We will also

collect pain data through Wheelchair User's Shoulder Pain Index. The Wheelchair User's Shoulder Pain Index is a 15-item self-report survey specifically designed to assess shoulder pain in wheelchair users during daily functional activities with a 10-point visual analog scale (86, 87). The Fatigue Severity Scale survey will include a 9-item measure of the severity of fatigue (76). The Fatigue Severity Scale has been evaluated for reliability, internal consistency, test-retest reliability, and validity for use in persons with SCI (25). The degree of agreement will be calculated by averaging the 7-point ordinal scale ranging from 1 (strongly disagree) to 7 (strongly agree) for the 9-item scores. Average score higher than 4 indicates significant fatigue (76). Deconditioning will be assessed by calculating the reduced capacity scores assessed through Leisure Time Physical Activity Questionnaire for People with Spinal Cord Injury (LTPAQ-SCI), a brief questionnaire used to measure PAs performed in addition to regular activities of daily living (77). The participant will report the number of days over the past 7 days when they performed PAs at low, medium, and high intensity PA levels. Next, they will report the number of minutes that they spent performing PAs at each of the intensity levels. The scale will be scored by calculating the total number of minutes of PAs performed at each intensity level (number of days of activity*number of minutes of activity), which will yield the total number of minutes of PA performed over the last 7 days (77).

Participant testing

Aim 1

The first visit can be at the participant's home or at Temple University, based on their choice. If their health care provider is MossRehab or Magee Rehabilitation then their first visit can be at their health care provider's facility. The participants will provide the informed consent and will answer questions about demographics, SCI, wheelchair information, and health and activity history (local environment and access to gym and recreation centers). The PA history will be assessed through the Physical Activity Recall Assessment for People with Spinal Cord injury (PARA-SCI). In addition, the participants will provide a Chronic Pain Grade questionnaire (72), Wheelchair User's Shoulder Pain Index (87), Fatigue Severity Scale (76), and a Leisure Time Physical Activity Questionnaire for People with SCI (77). The total time for the first visit will not exceed one hour and 15 minutes. If Dr. Ramos-Lamboy is their care provider then he will recommend them to continue their normal physical activity pattern. Otherwise Dr. Hiremath will recommend them to continue their normal physical activity pattern.

Participant will be asked to install a PAMS app and an AccuTracking app on their mobile phone if they choose to use their smartphone and run the apps. If they choose to use the research smartphone then the apps will be installed on it. The participants will be taught how to use and maintain PAMS. Using PAMS will involve placing the G-WRM in the case secured to the spokes of the wheelchair or a wheel-rotation sensor fixed to the wheelchair wheel and using the smartphone. Participants will also be asked to wear a provided smartwatch, only

taking off the watch for showering and swimming and charging the watch's battery. Participant will be asked to carry their smartphone or the study's smartphone (here onwards called as "the smartphone") with them as they normally would, trying to keep it within earshot most of the time. Participants will be asked to respond to questions when prompted. Participants may receive prompts either on the smartphone or on the smartwatch. The prompts will consist of audible beeps or vibration, and then a short survey will be presented on the phone or watch. The PAMS smartphone application will be running in the background of the smartphone and smartwatch continuously to determine when to prompt you, and to gather data on your motion. At the first meeting, participants will also be trained to recharge the smartphone and/or other devices once per day, typically at night. The first month of the study will involve collecting baseline physical activity levels.

Throughout the study, the smartphone will send information about how and when participants answer questions to the researchers using the phone's data connection. The phone will also send information about the status of the phone to the researchers. This phone status information will consist of phone's battery life remaining, the date and times the participant unlock the phone screen during the study, whether the phone is being moved, and when the phone may be in use. Information about the phone's location will also be collected, to better understand their movement patterns in their community. The phone may also send information about answers to questions on the smartwatch, the smartwatch's battery life remaining, and the whether the watch is being moved. No information about specific contacts for people they know, phone numbers of people they know or call or receive, or text messages will be used or gathered by the software. Within 24 hours of collecting any data, the smartphone will encrypt that information, so that if the smartphone were stolen, nobody would be able to use it. The encrypted data will be sent using a secure data transmission protocol to a password-protected server. Only the research team will be able to de-encrypt the participants' data, and once the participants' complete the study, their encrypted data will be taken off the Internet.

Researchers will expect that participant will:

- Charge the mobile phone nightly during the study and keep it with them as much as possible during the day.
- Wear their smartwatch at all times during the day when they can safely do so without immersing it water.
- Place the charger in the same place as where they recharge the smartphone each night, and charge the smartwatch nightly.
- Be careful with the smartwatch device and return it in good condition at the end of the study.
- Not use the smartwatch for anything other than the purpose of this study.

The researchers will show the participant how to setup the smartwatch with their phone, and then expect that they do not modify these settings in any way, including turning on or off any additional watch notifications or software.

Aim 2

Participants will answer survey questionnaires similar to those from their first visit (either at their home or at Temple University, MossRehab, or in the laboratory, based on the participant's choice). Participants will answer survey questionnaires similar to those from their first visit. If Dr. Ramos-Lamboy is their care provider then he will provide clinical recommendations on how much physical activity they should strive to obtain and how they might accomplish that for the second and third months of the testing. Otherwise Dr. Hiremath will provide the standard clinical recommendations on how much physical activity they should strive to obtain and how they might accomplish that for the second and third months of the testing. Dr. Hiremath, the PI, will work with Dr. Snethen to provide additional recommendations on how they can perform and attain regular PA levels in community settings. Participants will be trained on the new capability of the PAMS app, that will now provide daily feedback. The participants will perform three PAs including resting, wheelchair propulsion, and simulated arm-ergometry to learn how to interact with the PAMS app. Participants will also have the opportunity to set goals based on their interest and their clinician's recommendation to increase PAs. Participants will continue to use PAMS and answer questions on the phone and watch.

Aim 3

Participants will answer survey questionnaires similar to those from previous visits (either at their home or at Temple University, MossRehab, or in the laboratory, based on the participant's choice). Participants will answer survey questionnaires similar to those from the last visit. Providing personalized messages at specific times and contexts when activity is actually being performed and proactively congratulating the participants when they achieve their goals will enhance system engagement and thereby lead to behavior change. Dr. Hiremath, the PI, will re-discuss the PA recommendations provided in the second month. The research team will also indicate the way PAMS will provide PA recommendations based on the adaptive algorithms for the third month of the study. Participants will continue to use PAMS and provide answer questions on the phone and watch.

Post-study completion

Participant will answer survey questionnaires similar to the last meeting over the phone and return the PAMS equipment to the research team.

Data Analysis

Model development

We will develop models that incorporate each individual's PA level and clinician's PA level recommendation to provide a mobile phone application that helps set safe and highly personalized PA goals. PA guidelines for adults with disabilities recommend 150 mins of moderate or 75 mins of vigorous-intensity aerobic activity per week and, if possible, 2 days per week of muscle strengthening (2). Each individual will have a personalized model developed based on: individual's type, level, duration, and context of PAs obtained from the first month baseline PA level measurement, clinician's PA recommendation for each individual, and optimal level of PA that an individual can perform without increasing the risk of secondary conditions. We will simulate real-time recommendations in the laboratory by using the individualized models and the data collected from the first month to look for poor real-time recommendations during the second month of the testing, when the individuals' are only receiving passive feedback. The models will be revised in the last week of second month prior to updating them to the individuals' Android smartphone for the third month of testing. By adapting the goals in real-time based on the person's actual behavior, the model aims to keep the individual feeling positive and motivated.

The just-in-time intervention has the capability to interact with the person more than several times a day (57, 78) and will utilize PAMS to detect various wheelchair based PAs being performed by the user. The just-in-time recommendations will be simple, such as inquiring whether the participant is willing to continue performing the current PA for a slightly longer duration, thus making the participant more inclined to continue the PA. The model will also assess the context, level and the type of PA the person was compliant in performing when a just-in-time recommendation was provided. The list of contexts includes items such as home environment, accessible gymnasium, community centers, and trails. A key focus of this model will be to keep the suggestion actionable in the moment; tailored; appropriate for the context and hope that the model will change the habit of the people over time. In some ways, the model will incorporate aspects of operant conditioning, where providing positive feedback to the participants through encouraging and congratulating messages may lead to an increase in PA and/or sustain PA levels.

Letts et al. have indicated that peers and health service providers were preferred messengers for delivering PA information to individuals with SCI (79), which leads us to a clinician delivering PA information and providing PA recommendations. Clinician suggestion will involve performing PAs that are safe and target cardiovascular endurance, muscle strengthening, and flexibility. Individualized PA prescriptions will incorporate participant's PA interests (self-report) and wheelchair-based PAs detected by PAMS. PA prescriptions will be provided with recommendations for appropriate upper body strength training exercises (using free weights and/or elastic resistance bands), wheelchair pushing/cycling, or adaptive wheelchair sports (basketball, tennis, rugby, boxing, yoga, etc), and stretching exercises. Dr. Hiremath will work with Drs. Gretchen Snethen and Marlyn Ramos-Lamboy, and Mary Schmidt-Read to create an information booklet that will help individuals connect their PA interests to available resources in the community.

We will collect data through a short EMA on the smartwatch when the participant is not interested in performing additional PA. The feedback options for short EMAs on smartwatch will include choices such as not enough time, not interested, or other reasons. The information as to why a participant is not interested in performing additional PA may enlighten us with the barriers that exist with increasing PA levels in individuals who use wheelchairs in the community. We will also incorporate short EMAs that will inquire the participant about the type of PAs they are performing, when PAMS detects steady activity (>10mins), to be added to their PA interests.

<u>Model evaluation</u>

Univariate analysis will be performed to obtain a range of values and the central tendency for variables such as PA levels (Hyp. 1a) and sedentary behaviors (Hyp. 1b). Sedentary behavior will be assessed by the time duration of non-movement of individuals with SCI and not just the total duration of being seated in their wheelchairs. We hypothesize that the PA level of individuals with SCI in community will be low compared to the PA level recommendations for individuals with disabilities in general. Furthermore, the sedentary behavior of individuals with SCI will be high compared to the general population. Multiple regression analysis will be performed to assess a relationship between secondary conditions such as pain (scores), fatigue (scores), and deconditioning (reduced capacity scores) and PA levels (Hyp. 1c). We postulate that secondary conditions will be negatively correlated with the PA levels.

Repeated measures general linear model (GLM) analysis (82, 83) will be performed to assess the change in PA levels, sedentary behaviors (Hyp. 2, Hyp. 3a, 3b and 3c) and secondary conditions (Hyp. 3c). Repeated measures GLM analysis is appropriate when one or more factors are within-subjects factors. The results of within-subjects effects from the GLM procedure will indicate if the passive feedback and just-in-time adaptive feedback have a significant effect on the PA levels, sedentary behaviors, and secondary conditions over time. In addition, we will perform linear mixed model analysis (83) to develop a personal intercept (and maybe slope) for each participant compared to the mean intercept for each group (Hyp. 2, Hyp. 3a, 3b and 3c). The groups may be based on the results of the study where there is a clear data hierarchy based on the PA level performance for demographic characteristics such as gender or SCI (paraplegia vs. tetraplegia). Mixed effects model analysis will provide correct estimates of intervention (passive feedback and just-in-time adaptive feedback) and other fixed effects (within-subjects factor) in the presence of correlated data (each participant at different time points) that arise from a data hierarchy (group). We will perform non-parametric tests if the assumptions for parametric tests are not met. All statistical analysis will be performed using IBM SPSS Statistics software, with a statistical significance at an alpha level of 0.05.

e) Withdrawal of Subjects

All subjects will be reassured of their right to withdraw at any time from this study and will decide whether to sign the Human Consent Form approved by the Institutional Review Board of Temple University.

If the subject decides to stop the research study at any time then it will not be held against the subject. The subject will be asked to send any study related instruments back to the research team through a return postal package sent to the subject by the research team. If the subject stops being in the research study prior to its completion then the data collected to that point may not be removed from the study database.

In addition, the subject payment (as mentioned in Payment) will stop from the time point the subject decides to stop the participation in the study.

The person in charge of the research study or the sponsor can remove the subject from the research study without their approval. Possible reasons for removal include failure to follow instructions of the research staff or if the principal investigator decides that the research study is no longer in the subject's best interest. The sponsor can also end the research study early.

f) Privacy & Confidentiality

This study will use subjects' protected health information, and will require subjects to sign a HIPAA Authorization Form. All research staff will be trained in privacy regulations and procedures.

All paper documents will be stored in locked cabinets and electronic documents will be stored on password protected computers in the Personal Health Informatics and Rehabilitation Engineering (PHIRE) lab or investigators' offices for further data analysis. All subjects will be identified using an alpha-numeric identification system (such as JITA001 – Just-in-time adaptive Subject #1). Any personal, health, or medical information recorded will be protected according to HIPAA regulations. Access to the PHIRE lab spaces and investigators' offices requires keys. Privacy measures in place for participants during the research activities (consent, answering surveys) will consist of consent in a private room. All consent forms will be stored for at least three years following the termination of a given project in the locked cabinets in the investigators or lab space.

The physical activity and survey data (without HIPAA identifiers) collected using the PAMS application will be directly uploaded to a secure server. All precautions will be taken to keep this data secure. No information about specific contacts for people the participants' know, phone numbers of people they know or call or receive, or text messages will be used or gathered by the software. Within 24 hours of collecting any data, the smartphone will encrypt that information, so that if the smartphone were stolen, nobody would be able to use it. The encrypted data will be sent using a secure data transmission protocol to a password-protected server. Only the research team will be able to de-encrypt participants' data, and

once the participants complete the study, their encrypted data will be taken off the Internet.

We are collecting location data via a smartphone application (Accutracking.com). According to the company's Privacy Policy, the GPS data is stored on their server and may be reviewed by them for troubleshooting or support services. In those instances, even though AccuTracking may potentially see location information of our study participants they will not have the names or other identifying information of study participants, and their point of contact will be the study investigators. Furthermore, all location data are deleted automatically from the AccuTracking system after a month. In addition, their Privacy Policy states that they will not lend, sell or otherwise make available users' personal information without their permission, unless required by law, subpoenas, warrant, or court order (http://www.accutracking.com/privacypolicy.html). Once the tracking is complete, the location data will be downloaded via a secure internet connection onto a HIPAA-approved server. The data will be only be accessible to research personnel, who, prior to access to any study-related information, will be required to have the necessary training to protect the security and confidentiality of identifiable information.

All software installed on the participants' smartphone or the study's smartphone will be removed when they remove the app, which the researchers will help them to do. The information left on the smartwatch will be wiped clean once the participants hand the watch back to the researchers.

The results of the study will not be shared with the subject or others.

In case of emergency during the experiment, 911 will be called.

All research personnel for the study will be trained in order to show respect to and interact with participants in the way that the subjects will feel at ease with the research situation in response to questions, examinations and experimental procedures.

9) Risks to Subjects

The probability and magnitude of harm or discomfort anticipated in the research is not greater than during the performance of routine PAs and activities of daily living. It is anticipated that some participants may be inconvenienced by the amount of time necessary to complete the study. There is potential for interruption of their day when the participants are prompted for information on their phone or the smartwatch, possibly causing annoyance, or frustration. Participants are expected and urged to ignore interruptions that could cause a safety problem (such as while driving) or that might jeopardize their work. Participants may also experience muscle fatigue or pain while performing additional PAs. Participants will have the opportunity to discuss these issues with the PI. Participants may discontinue the study at any time. To maintain confidentiality, records will be de-identified by assigning a case number and the

information linking the case number to the participant's identity will be stored in a separate, locked location within the PHIRE. Access to these files are restricted to the PI and the associated research staff working on the project. In addition, all research staff will be trained in privacy regulations and procedures.

The PA and EMA data (without HIPAA identifiers) collected using the PAMS application will be directly uploaded to the secure virtual machine research servers at Dr. Intille's research groups. All precautions, as mentioned above, will be taken to keep this data secure. The data about mobility collected through the AccuTracking application will be sent to a secure online database which in turn will be downloaded to our secure HIPAA-approved servers at Temple University (also please refer to the section on Privacy and Confidentiality for more information).

Taking part in this research study may lead to transportation costs to the participants, which can be covered by their participant stipend. Participants will be informed that the use of the software will transfer a small amount of data to the research server as well as drain their smartphone battery somewhat faster than usual if they choose to use their own smartphone. The amount of data transmitted will be approximately the equivalent of sending a few pictures from the camera each day.

10) Potential Benefits to Subjects

There is no direct benefit to study participants. However, possible benefits to others include the resulting scientific knowledge that will provide foundation to create novel physical activity monitors for individuals with spinal cord injury. This research could lead to less burdensome and more accurate ways to measure physical activity in the community. The research might also provide information that helps other researchers learn how to create wearable technology that can learn about and track physical activity behavior and be used to create new health and assistive interventions.

11) Costs to Subjects

Research subjects will not be responsible for any costs related to participating in the research.

12) Informed Consent

Investigators will follow "INVESTIGATOR GUIDANCE: Informed Consent (HRP-802)."

Written informed consents will be provided by all subjects. The consent process will occur in a private area at the testing site at the time of testing. The consent form will be discussed with all subjects by an investigator, and the subject will be

offered chance to consent at the conclusion of this discussion. Should the potential subject wish additional time to consider whether to consent, they will be given as much time as they desire. All subjects will receive a copy of the consent from for their records. All investigators involved in the consenting process have been identified on the IRB application as investigators who will have access to subject data and the consent process. Verbal discussion will ensure subjects understand each consent topic as it is discussed, and subjects will be advised that their signature (as well as initials on each page of the consent form) indicates their understanding and consent. It is reinforced throughout the consent form that subjects are free to ask questions at any stage of the study, including before, during, or after the consent process.

Investigators will follow “INVESTIGATOR GUIDANCE: Documentation of Informed Consent (HRP-803).”

13) Vulnerable Populations

All subjects admitted to the study will be legal adults who understand the study and freely provide written content. Study subjects will not include minors, pregnant women, prisoners, or those with cognitive impairments or who are not able to provide consent.

References

1. Martin SB, Morrow JR, Jackson AW, Dunn AL. Variables related to meeting the CDC/ACSM physical activity guidelines. Medicine and Science in Sports and Exercise. 2000;32(12):2087-92.
2. U.S. Department of Health and Human Services. Physical Activity: Overview. 2012.
3. Washburn R, Hedrick BN. Descriptive epidemiology of physical activity in university graduates with locomotor disabilities. International Journal of Rehabilitation Research. 1997;20(3):275-87.
4. Tasiemski T, Kennedy P, Gardner BP, Taylor N. The association of sports and physical recreation with life satisfaction in a community sample of people with spinal cord injuries. NeuroRehabilitation. 2005;20:253-65.
5. Fernhall B, Heffernan K, Jae SY, Hedrick B. Health implications of physical activity in individuals with spinal cord injury: a literature review. Journal of Health and Human Services Administration. 2008;30(4):468-502.
6. Dearwater SR, Laporte RE, Robertson RJ, Brenes G, Adams LL, Becker D. Activity in spinal cord injured patients: an epidemiological analysis of metabolic parameters. Medicine and Science in Sports and Exercise. 1986;18(5):541-44.
7. Tasiemski T, Bergström E, Savic G, Gardner BP. Sports recreation and employment following spinal cord injury - a pilot study. Spinal Cord. 2000;38(3):173-84.
8. Centers for Disease Control and Prevention (CDC). National Center for Health Statistics DATA 2010 [Internet database] Hyattsville, MD: CDC; 2010 http://wondercdcgov/data2010/focushtm.
Accessed 2011, January 10.
9. Glaser RM, Janssen TWJ, Suryaprasad AG, Gupta SC, Mathews T. The Physiology of Exercise. In: Apple DF, editor. Physical Fitness: A Guide for Individuals with Spinal Cord Injury. Washington, DC: Department of Veterans Affairs; 1996.
10. Warms C. Physical activity measurement in persons with chronic and disabling conditions. Family Community Health. 2006;29(S1):78S-88S.
11. Ginis KM, Latimer AE, Hicks AL, Craven BC. Development and evaluation of an activity measure for people with spinal cord injury. Medicine and Science in Sports and Exercise. 2005;37(7):1099-111.
12. Washburn RA, Zhu W, McAuley E, Frogley M, Figoni SF. The physical activity scale for individuals with physical disabilities: development and evaluation. Archives of Physical Medicine and Rehabilitation. 2002;83(2):193-200.
13. Tolerico ML, Ding D, Cooper RA, Spaeth DM, Fitzgerald SG, Cooper R, et al. Assessing mobility characteristics and activity levels of manual wheelchair users. Journal of Rehabilitation R and D. 2007;44(4):561-72.
14. Sonenblum SE, Sprigle S, Caspall J, Lopez R. Validation of an accelerometer-based method to measure the use of manual wheelchairs. Medical Engineering and Physics. 2012;34:781-86.
15. Conger SA, Scott SN, Bassett DR. Predicting energy expenditure through hand rim propulsion power output in individuals who use wheelchairs. British Journal of Sports Medicine. 2014;48:1048-53.
16. Hiremath SV, Ding D, Cooper RA. Development and evaluation of a gyroscope based wheel rotation monitor for manual wheelchair users. Spinal Cord Medicine. 2013;36(4):347-56.
17. Hiremath SV, Ding D, Farringdon J, Cooper RA. Predicting energy expenditure of manual wheelchair users with spinal cord injury using a multi-sensor based activity monitor. Archives of Physical Medicine and Rehabilitation. 2012;93(11):1937-43.
18. Kiuchi K, Inayama T, Muraoka Y, Ikemoto S, Uemura O, Mizuno K. Preliminary study for the assessment of physical activity using a triaxial accelerometer with a gyro sensor on the upper limbs of subjects with paraplegia driving a wheelchair on a treadmill. Spinal Cord. 2014;52:556-63.
19. Nightingale TE, Walhin J-P, Thompson D, Bilzon JL. Predicting Physical Activity Energy Expenditure in Manual Wheelchair Users. Medicine and Science in Sports and Exercise. 2014;46(9):1849-58.
20. Hiremath SV. Physical Activity Monitoring System for Manual Wheelchair Users: Doctoral

Dissertation, University of Pittsburgh; 2013.
21. Hiremath SV, Intille SS, Kelleher A, Cooper RA, Ding D. Detection of physical activities using a physical activity monitor system for wheelchair users. Medical Engineering and Physics. 2015;37(1):68-76.
22. Hiremath SV, Intille SS, Kelleher A, Cooper RA, Ding D. Estimation of energy expenditure for wheelchair users using a Physical Activity Monitoring System. Archives of Physical Medicine and Rehabilitation. Under Review.
23. Paralyzed Veterans of America Consortium for Spinal Cord Medicine. Preservation of upper limb function following spinal cord injury: A clinical practice guideline for health-care professionals. Journal of Spinal Cord Medicine. 2005;28(5):434–70.
24. Turner JA, Cardenas DD, Warms CA, McClellan CB. Chronic pain associated with spinal cord injuries: a community survey. Archives of Physical Medicine and Rehabilitation. 2001;82(4):501-8.
25. Anton HA, Miller WC, Townson AF. Measuring fatigue in persons with spinal cord injury. Archives of Physical Medicine and Rehabilitation. 2008;89(3):538-42.
26. Rimmer JH, Schiller W, Chen M-D. Effects of disability-associated low energy expenditure deconditioning syndrome. Exercise and Sport Sciences Reviews. 2012;40(1):22-9.
27. Bond DS, Thomas JG, Raynor HA, Moon J, Sieling J, Trautvetter J, et al. B-mobile-a smartphone based intervention to reduce sedentary time in overweight/obese individuals: A within-subjects experimental trial. 2014.
28. Intille SS. A new research challenge: persuasive technology to motivate healthy aging. Information Technology in Biomedicine, IEEE Transactions on. 2004;8(3):235-7.
29. Durstine JL, Painter P, Franklin BA, Morgan D, Pitetti KH, Roberts SO. Physical activity for the chronically ill and disabled. Sports Medicine. 2000;30(3):207-19.
30. Hoenig H, Landerman LR, Shipp KM, George L. Activity restriction among wheelchair users. Journal of American Geriatrics Society. 2003;51(9):1244-51.
31. Rimmer JH. Use of the ICF in identifying factors that impact participation in physical activity/rehabilitation among people with disabilities. Disability and Rehabilitation. 2006;28(17):1087 – 95.
32. Jacobs PL, Nash MS, Rusinowski JW. Circuit training provides cardiorespiratory and strength benefits in persons with paraplegia. Medicine and Science in Sports and Exercise. 2001;33:711-7.
33. Rimmer JH, Yamaki K, Davis BM, Wang E, Vogel LC. Obesity and Overweight Prevalence Among Adolescents With Disabilities. Preventing Chronic Disease. 2011;8(2):1-6.
34. Rimmer JH, Shenoy SS. Impact of Exercise on Targeted Secondary Conditions. Field MJ, Jette AM, Martin L, editors. Washington, DC: National Academy of Sciences; 2006.
35. Center NSCIS. Spinal cord injury facts and figures at a glance. The journal of spinal cord medicine. 2013;36(1):1.
36. Buchholz AC, McGillivray CF, Pencharz PB. Physical Activity Levels Are Low in Free-Living Adults with Chronic Paraplegia. Obesity research. 2003;11(4):563-70.
37. Collins EG, Gater D, Kiratli J, Butler J, Hanson K, Langbein WE. Energy cost of physical activities in persons with spinal cord injury. Medicine and science in sports and exercise. 2010;42(4):691-700.
38. Organization WH. Obesity: preventing and managing the global epidemic: World Health Organization; 2000.
39. Jakicic JM, Tate DF, Lang W, Davis KK, Polzien K, Rickman AD, et al. Effect of a stepped-care intervention approach on weight loss in adults: a randomized clinical trial. Journal of the American Medical Association. 2012;307(24):2617-26.
40. Coons MJ, DeMott A, Buscemi J, Duncan JM, Pellegrini CA, Steglitz J, et al. Technology Interventions to Curb Obesity: A Systematic Review of the Current Literature. Current cardiovascular risk reports. 2012;6:120-34.
41. Alhassan S, Kim S, Bersamin A, King A, Gardner C. Dietary adherence and weight loss success among overweight women: results from the A to Z weight loss study. International Journal of Obesity. 2008;32:985-91.
42. Baker RC, Kirschenbaum DS. Self-monitoring may be necessary for successful weight control. Behavior Therapy. 1993;24:377–94.

43. Wing R, Phelan S. Long-term weight loss maintenance. American Journal of Clinical Nutrition.
2005;82:2225–55.
44. Shuger SL, Barry VW, Sui X, McClain A, Hand GA, Wilcox S, et al. Electronic feedback in a diet- and physical activity-based lifestyle intervention for weight loss: a randomized controlled trial. International Journal of Behavioral Nutrition and Physical Activity. 2011;8(41).
45. Spring B, Duncan JM, Janke EA, Kozak AT, McFadden HG, Demott A, et al. Integrating technology into standard weight loss treatment a randomized controlled trial. JAMA Internal Medicine. 2013;173(2):105-11.
46. Rimmer JH, Riley BB, Rubin SS. A new measure for assessing the physical activity behaviors of persons with disabilities and chronic health conditions: the Physical Activity and Disability Survey. American Journal of Health Promotion. 2001;16(1):34-45.
47. Fix AJ, Daughton D. Human activity profile: Professional manual: Psychological Assessment Resources; 1988.
48. Tudor-Locke CE, Myers AM. Challenges and opportunities for measuring physical activity in sedentary adults. Sports Medicine. 2001;31(2):91-100.
49. Coulter EH, Dall PM, Rochester L, Hasler JP, Granat MH. Development and validation of a physical activity monitor for use on a wheelchair. Journal of Spinal Cord. 2011;49:445-50.
50. García-Massó X, Serra-Añó P, García-Raffi L, Sánchez-Pérez E, López-Pascual J, Gonzalez L. Validation of the use of Actigraph GT3X accelerometers to estimate energy expenditure in full time manual wheelchair users with spinal cord injury. Spinal Cord. 2013;51(12):898-903.
51. Hicks A, Martin K, Ditor D, Latimer A, Craven C, Bugaresti J, et al. Long-term exercise training in persons with spinal cord injury: effects on strength, arm ergometry performance and psychological well-being. Spinal cord. 2003;41(1):34-43.
52. Abel T, Platen P, Vega SR, Schneider S, Strüder H. Energy expenditure in ball games for wheelchair users. Spinal Cord. 2008;46(12):785-90.
53. Abel T, Kröner M, Rojas VS, Peters C, Klose C, Platen P. Energy expenditure in wheelchair racing and handbiking-a basis for prevention of cardiovascular diseases in those with disabilities. European Journal of Cardiovascular Prevention & Rehabilitation. 2003;10(5):371-6.
54. Roy JL, Menear KS, Schmid MM, Hunter GR, Malone LA. Physiological responses of skilled players during a competitive wheelchair tennis match. The Journal of Strength & Conditioning Research. 2006;20(3):665-71.
55. Barfield J, Malone LA, Coleman TA. Comparison of heart rate response to tennis activity between persons with and without spinal cord injuries: implications for a training threshold. Research quarterly for exercise and sport. 2009;80(1):71-7.
56. Maki KC, Langbein WE, Reid-Lokos C. Energy cost and locomotive economy of handbike and rowcycle propulsion by persons with spinal cord injury. Journal of rehabilitation research and development. 1995;32:170-.
57. Spruijt-Metz D, Nilsen W. Dynamic models of behavior for just-in-time adaptive interventions. IEEE Pervasive Computing. 2014 (3):13-7.
58. Wadden TA, Webb VL, Moran CH, Bailer BA. Lifestyle modification for obesity new developments in diet, physical activity, and behavior therapy. Circulation. 2012;125(9):1157-70.
59. Dunton GF, Dzubur E, Kawabata K, Yanez B, Bo B, Intille S. Development of a smartphone application to measure physical activity using sensor-assisted self-report. Frontiers in public health. 2014;2.
60. Intille SS, Albinali F, Mota S, Kuris B, Botana P, Haskell WL, editors. Design of a wearable physical activity monitoring system using mobile phones and accelerometers. Conference proceedings: Annual International Conference of the IEEE Engineering in Medicine and Biology Society IEEE Engineering in Medicine and Biology Society Annual Conference; 2010.
61. Reynolds GS. A primer of operant conditioning.(Rev ed). 1975.
62. Ginis KAM, Latimer AE, Arbour-Nicitopoulos KP, Bassett RL, Wolfe DL, Hanna SE. Determinants of physical activity among people with spinal cord injury: a test of social cognitive theory. Annals of Behavioral Medicine. 2011;42(1):127-33.

63. Wongsirikul N. Field-based usability study of physical activity monitoring and sharing system for manual wheelchair users with spinal cord injury [Master's Thesis]: University of Pittsburgh; 2014.
64. Medicine ACoS. ACSM's guidelines for exercise testing and prescription: Lippincott Williams & Wilkins; 2013.
65. Ledger D, McCaffrey D. How the Science of Human Behavior Change Offers the Secret to Long-Term Engagement. Endeavour Partners LLC; 2014.
66. Ledger D. The Future of Activity Trackers (Part 3): The Secret to Long-Term Engagement. Endeavour Partners LLC; 2014; Available from: http://endeavourpartners.net/the-future-of-activity-trackers-part-3-the-secret-to-long-term-engagement/.
67. Boninger ML, Souza AL, Cooper RA, Fitzgerald SG, Koontz AM, Fay BT. Propulsion patterns and pushrim biomechanics in manual wheelchair propulsion. Archives of physical medicine and rehabilitation. 2002;83(5):718-23.
68. Brose SW, Boninger ML, Fullerton B, McCann T, Collinger JL, Impink BG, et al. Shoulder ultrasound abnormalities, physical examination findings, and pain in manual wheelchair users with spinal cord injury. Archives of physical medicine and rehabilitation. 2008;89(11):2086-93.
69. Heath GW, Fentem PH. Physical activity among persons with disabilities--a public health perspective. Exercise and sport sciences reviews. 1996;25:195-234.
70. Batavia AI. Of wheelchairs and managed care. Health Affairs. 1999;18(6):177.
71. Karmarkar A, Chavez E, Cooper RA. Technology for successful aging and disabilities. The Engineering Handbook of Smart Technology for Aging, Disability and Independence. 2008:1000.
72. Von Korff M, Ormel J, Keefe FJ, Dworkin SF. Grading the severity of chronic pain. Pain. 1992;50(2):133-49.
73. Smith BH, Penny KI, Purves AM, Munro C, Wilson B, Grimshaw J, et al. The Chronic Pain Grade questionnaire: validation and reliability in postal research. Pain. 1997;71(2):141-7.
74. Penny KI, Purves AM, Smith BH, Chambers WA, Smith WC. Relationship between the chronic pain grade and measures of physical, social and psychological well-being. Pain. 1999;79(2):275-9.
75. Underwood MR, Barnett AG, Vickers MR. Evaluation of Two Time-Specific Back Pain Outcome Measures. Spine. 1999;24(11):1104-12.
76. Krupp LB, LaRocca NG, Muir-Nash J, Steinberg AD. The fatigue severity scale: application to patients with multiple sclerosis and systemic lupus erythematosus. Archives of Neurology. 1989;46(10):1121-3.
77. Ginis KAM, Phang SH, Latimer AE, Arbour-Nicitopoulos KP. Reliability and validity tests of the leisure time physical activity questionnaire for people with spinal cord injury. Archives of Physical Medicine and Rehabilitation. 2012;93(4):677-82.
78. Spruijt-Metz D, Hekler E, Saranummi N, Intille S, Korhonen I, Nilsen W, et al. Building new computational models to support health behavior change and maintenance: new opportunities in behavioral research. Translational Behavioral Medicine.1-12.
79. Letts L, Ginis KAM, Faulkner G, Colquhoun H, Levac D, Gorczynski P. Preferred methods and messengers for delivering physical activity information to people with spinal cord injury: A focus group study. Rehabilitation psychology. 2011;56(2):128.
80. Ginis KA, Latimer AE, Hicks AL, Craven BC. Development and evaluation of an activity measure for people with spinal cord injury. Medicine and Science in Sports and Exercise. 2005;37(7):1099–111.
81. Latimer AE, MartinGinis KA, Craven BC, Hicks AL. The physical activity recall assessment for people with spinal cord injury: validity. Medicine & Science in Sports & Exercise. 2006;38(2):208-16.
82. UCLA: Statistical Consulting Group. Repeated Measures Analysis with SPSS. 2015 [February 10, 2015]; Available from: http://www.ats.ucla.edu/stat/spss/seminars/Repeated_Measures/.
83. Seltman HJ. Experimental design and analysis. Online at: http://www stat cmu edu/, hseltman/309/Book/Book pdf. 2012.
84. Zhou C, Ludford P, Frankowski D, Terveen L.. How Do People’s Concepts of Place Relate to Physical Locations? INTERACT Conference. Rome, Italy. 2005.

85. PanoBike LLC, Speed and Cadence Sensor. http://www.topeak.com/products/PanoBikeAndRideCase/speed_cadence_sensor
86. Curtis KA, Roach KE, Applegate EB, et al. Reliability and validity of the Wheelchair User's Shoulder Pain Index (WUSPI). Paraplegia. 1995;33:595– 601.
87. Curtis KA, Roach KE, Applegate EB, et al. Development of the Wheelchair User's Shoulder Pain Index (WUSPI). Paraplegia. 1995;33: 290–293.
