# Supplementary material for: Mobile health-based physical activity intervention for individuals with spinal cord injury in the community: A pilot study
Source: PLoS One. 2019 Oct 15;14(10):e0223762. doi: 10.1371/journal.pone.0223762 (PMC6793862; doi:10.1371/journal.pone.0223762)
Supplement: S2 File — (PDF) [file pone.0223762.s002.pdf]

Demographics and Basic Information

Date: ___/___/______

Gender: ❑ Male (1) ❑ Female (0)

Age: ________

Body weight: ________lbs

Height: ________feet ________inches

SCI Level ________________________

1. **Completeness of Injury:** ❑ Complete ❑ Incomplete

2. **Date of Injury Onset:** ___/___/______

3. **Ethnic Origin:**
   ❑ African American (1) ❑ Asian American (2) ❑ Caucasian (3)
   ❑ Hispanic (4) ❑ Native American (5) ❑ Other (6):______________

4. **Manual Wheelchair Make (brand):**
   ❑ Action/Invacare ❑ Pride ❑ Everest and Jennings
   ❑ Sunrise/Quickie ❑ Kuschall ❑ TiLite/TiSport
   ❑ Otto Bock ❑ Other (please specify): _____________________

5. **Manual Wheelchair Model**: _________________

6. **Diameter of your wheelchair's wheel in inches:** _________________

7. **When did you start using a manual wheelchair**: ____/____/____ (mm/dd/year)

8. **Which is your dominant hand?** ❑ Right ❑ Left

9. **Are you an athlete?** ❑ Yes ❑ No

10. **Do you smoke?** ❑ Yes ❑ No

11. **Have you had or do you presently have any of the following conditions?**
    ❑ High blood pressure ❑ Seizures ❑ Lung disease
    ❑ Fainting or dizziness ❑ Diabetes ❑ High cholesterol
    ❑ Shortness of breath at rest or with mild exertion
    ❑ Unusual fatigue or shortness of breath with usual activities

12. **Do you follow any specific dietary intake plan?** ❑ Yes ❑ No

13. **In general how do you feel about your nutritional habits?**
    ❑ Excellent ❑ Very good ❑ Good
    ❑ Fair ❑ Poor

Physical Activity Information

1. **What is the approximate distance you propel your wheelchair on a typical day?**
   - ❑ ______miles per day
   - ❑ ______miles per week (include weekdays and weekends)
   - ❑ Don't know/Not sure

2. **During the past month, other than your regular job and propelling your wheelchair, did you participate in any physical activities or exercises? (Check all that apply)**

**Activity Type**	**Check (✓)**	**Frequency (number of times per week)**	**Average duration of each exercise session (minutes)**
Handcycling			
Wheelchair Basketball			
Wheelchair Tennis			
Wheelchair Rugby			
Arm-ergometry			
Swimming			
Weights			
Resistance Band			
Sled Hockey			
Other:			
Other:			
Other:			

3. **In general, how do you rate your fitness level?**
   - ❑ Excellent ❑ Very good ❑ Good
   - ❑ Fair ❑ Poor

4. **Do you have an access to a gym?** ❑ Yes ❑ No
   a. **If yes is the gym accessible?** ❑ Yes ❑ No
   Comments: ____

5. **Do you have an access to a recreation center?** ❑ Yes ❑ No
   a. **If yes is the recreation center accessible?** ❑ Yes ❑ No
   Comments: ____

Smart Phone Use Information

1. **Do you have a smart phone (i.e., a phone that can access internet)?**
   ❑ If no, please provide a reason (check all that apply, and skip the rest of questions from the Smart Phone Use Information questionnaire)
   - ❑ Cost ❑ I've tried, but I found it difficult to use
   - ❑ I do not need other features except calls.
   - ❑ Other _________

   ❑ If yes, (check all that apply):
   - ❑ IPhone ❑ Blackberry ❑ Motorola android
   - ❑ HTC android ❑ Samsung galaxy ❑ Other _________

2. **Does your phone have touch screen capability?**
   ❑ Yes
   - ❑ Very easy to use ❑ Somewhat easy to use ❑ A little difficult to use
   - ❑ Difficult to use

   ❑ No

3. **How long have you been using a smart phone?**
   - ❑ Less than a month ❑ 1-6 month ❑ 6 month – a year
   - ❑ 1-2 year ❑ 2-3 year ❑ More than 3 years

4. **What provider that you're using right now for your smart phone?**
   - ❑ Verizon ❑ AT&T ❑ T-Mobile
   - ❑ Sprint ❑ Virgin ❑ Cricket
   - ❑ Other ___________________

5. **Please state your average hours of smart phone use per day (including phone calls, internet browsing, email etc.)?**
   - ❑ Less than 1 hour ❑ 1-2 hours ❑ 2-4 hours
   - ❑ 4-6 hours ❑ More than 6 hours

6. **When you use your smart phone, what functions do you usually use? (Choose all that apply)**
   - ❑ Browsing internet ❑ Entertaining yourself ❑ Accessing social networks
   - ❑ Accessing email ❑ Text messaging ❑ Other ___________________

7. **On a scale of 1-5 (1 being low and 5 being high), how fluent do you regard yourself as a smart phone user?**
   1 2 3 4 5

8. **On a scale of 1-5, how essential is a smart phone to you?**
   1 2 3 4 5

9. **On a scale of 1-5, how satisfied are you with your current smart phone?**
   1 2 3 4 5

10. **In your opinion, what do you miss from your smart phone (check all that apply)?**
    - ❑ Bigger screen size ❑ Bigger keyboard ❑ Bigger font size
    - ❑ Simplicity of using it ❑ None of the above ❑ Other _______

FATIGUE SEVERITY SCALE (FSS)

Date ______________________________ Name __________________________________

Please circle the number between 1 and 7 which you feel best fits the following statements. This refers to your usual way of life within the last week. 1 indicates "strongly disagree" and 7 indicates "strongly agree."

Read and circle a number.	Strongly Disagree → Strongly Agree
1. My motivation is lower when I am fatigued.	1 2 3 4 5 6 7
2. Exercise brings on my fatigue.	1 2 3 4 5 6 7
3. I am easily fatigued.	1 2 3 4 5 6 7
4. Fatigue interferes with my physical functioning.	1 2 3 4 5 6 7
5. Fatigue causes frequent problems for me.	1 2 3 4 5 6 7
6. My fatigue prevents sustained physical functioning.	1 2 3 4 5 6 7
7. Fatigue interferes with carrying out certain duties and responsibilities.	1 2 3 4 5 6 7
8. Fatigue is among my most disabling symptoms.	1 2 3 4 5 6 7
9. Fatigue interferes with my work, family, or social life.	1 2 3 4 5 6 7

VISUAL ANALOGUE FATIGUE SCALE (VAFS)

Please mark an "X" on the number line which describes your global fatigue with 0 being worst and 10 being normal.

0	1	2	3	4	5	6	7	8	9	10
